# Antihistamine effects and safety of fexofenadine: a systematic review and Meta-analysis of randomized controlled trials

**DOI:** 10.1186/s40360-019-0363-1

**Published:** 2019-11-29

**Authors:** Cheng-zhi Huang, Zhi-hui Jiang, Jian Wang, Yue Luo, Hua Peng

**Affiliations:** 1 Department of Otolaryngology Head and Neck Surgery, General Hospital of Southern Theatre Command of PLA, Guangzhou, 510010 China; 2 Department of Otolaryngology Head and Neck Surgery, Shenzhen University General Hospital, Shenzhen, 518055 China; 3 Department of Pharmacy, General Hospital of Southern Theatre Command of PLA, Guangzhou, 510010 China; 4 Integrated Hospital of Traditional Chinese Medicine, Southern Medical University, Guangzhou, 510315 China; 5 Cancer Center, Southern Medical University, Guangzhou, 510315 China

**Keywords:** Fexofenadine, Antihistamines, Randomized controlled trial, Systematic review, Meta-analysis

## Abstract

**Background:**

As a new generation antihistamine, fexofenadine has been widely used in allergic diseases. However, there is still a lack of collective evidence regarding the antihistamine effects and safety profiles of fexofenadine relative to other antihistamine drugs and placebo. Therefore, we aimed to systematically evaluate the antihistamine effects and safety of fexofenadine.

**Methods:**

An electronic literature search of randomized controlled trials (RCTs) was performed using Embase, Cochrane and PubMed from establishment to January 1st, 2018. RCTs comparing the antihistamine effects or safety (adverse events, sedative effects, and cognitive/psychomotor function) of fexofenadine with either other antihistamines or placebo for healthy subjects and patients with allergy were selected.

**Results:**

Fifty-one studies of 14,551 participants met the inclusion criteria. When compared with the first-generation antihistamines, fexofenadine produced significantly lower adverse events frequency (OR = 0.446; 95% CI: 0.214 to 0.929, *P* = 0.031), significantly lower sedative effects frequency (OR = 0.265; 95% CI: 0.072 to 0.976, *P* = 0.046) and significantly less change of all cognitive/psychomotor function. When compared with the second-generation antihistamines, fexofenadine produced significantly marginal sedative effects (OR = 0.59; 95% CI, 0.38 to 0.93; *P* = 0.02) and significantly less change of most of the cognitive/psychomotor function. When compared with placebo, fexofenadine produced more significant antihistamine effects.

**Conclusions:**

Fexofenadine has a positive antihistamine effect, which is probably no worse than the second-generation antihistamines. Fexofenadine probably has a favorable safety profile, which is more likely better than that of the first-generation antihistamines. There is lack of data to support that fexofenadine has a better overall safety profile compared to the second-generation antihistamines, however, some presently available evidence on sedative effects and certain aspects of cognitive/psychomotor function favors fexofenadine. Therefore, fexofenadine may be worthy of recommendation for safety related workers.

## Background

The incidence of allergic diseases such as allergic rhinitis (AR), allergic asthma (AA), chronic idiopathic urticaria (CIU) and atopic dermatitis (AD) has continued to rise over the past several decades, affecting a large number of people worldwide [1]. Symptoms such as itching, sneezing, rhinorrhea and rhinobyon caused by allergic diseases usually lower the quality of life [2]. In fact, millions of people have been reported to experience physical impairments and reductions in quality of life, as well as economic burdens, derived from allergic diseases and its associated comorbidities [3]. Antihistamines have been widely used as a first-line drug in the treatment of allergic diseases. The first-generation antihistamines were no longer recommended because of their side effects including hepatotoxicity, cardiotoxicity, sedative effects, anticholinergic effects and lack of selectivity for the H1-receptor [4]. The second-generation antihistamines have replaced the first-generation antihistamines as commonly used drug in the treatment of allergic diseases because of their modest sedative effects and more significant and persistent curative effect compared with the first-generation antihistamines [4]. However, some of the second-generation antihistamines, such as terfenadine and astemizole, are rarely used because of their apparent cardiotoxicity [5]. As a new generation antihistamine and an active metabolite of terfenadine - a highly selective H1 antagonist, fexofenadine has positive antihistamine effects [6]. In addition, fexofenadine has no cardiotoxicity and minimal adverse effects on liver because only about 5% dosage of fexofenadine is metabolized by liver. As the substrate of P-glycoprotein, fexofenadine that is difficult to pass the blood-brain barrier may have no sedative effect and other central nervous functions [7]. To date, there is still a lack of collective evidence regarding the antihistamine effects and safety profiles of fexofenadine relative to other antihistamine drugs and placebo. As such, the aim of this study was to analyze the antihistamine effects and safety of fexofenadine in healthy subjects and patients with allergic diseases including AR, AA, CIU, and AD when compared with other antihistamines or placebo.

## Methods

### Eligibility criteria

Randomized controlled trials (RCTs) involving comparisons of antihistamine effects and safety of fexofenadine with either other antihistamines or placebo were included. Participants in these RCTs including healthy volunteers and patients with indications requiring treatment of antihistamines.

### Search strategy

A systematic literature search of Embase, Cochrane and PubMed were conducted with no limits on language, publication year, or publication status. The date of the last search was January 1st, 2018. The search term strategy was as follows: “fexofenadine”, “telfast”, “allegra”, AND “health*”, “allerg*”, “rhinitis”, “cold”, “asthma”, “Kimura”, “atopic”, “dermatitis”, “atopy”, “urticaria”, OR “effec*”, “antihistami*”, “skin”, “wheal”, “flare”, “safe*”, “drows*”, “sleep*”, “somnolence”, “alert*”, and “sedat*”. References of included studies and additional sources were examined to reduce the search bias.

### Study selection process

Endnote X7 program was used to eliminate duplicate references. The first round of screening was performed by reading title and abstract, the second round of screening was eligibility evaluation from the full text. All operations were performed by 2 separate reviewers and checked by the principal investigator. Any disagreements were resolved by discussion.

### Data extraction

For each included literature, the following data were extracted: first author, date of publication, mean age, gender, number of subjects lost to follow-up, study type, participant, number of subjects receiving fexofenadine, comparators, number of subjects receiving comparators, the dose of fexofenadine and comparators, study duration, outcome measures. If more than 1 dose of fexofenadine or more than 1 type of other generation antihistamines were assessed, we selected the one considered more effective and safer by the authors of the paper as the assessment of antihistamine effects and cognitive/psychomotor function, and combined all doses of fexofenadine or the same generation antihistamines for the evaluation of adverse events (AE) frequency and sedative effects frequency. When data were not available in certain papers, the authors were contacted directly by e-mail. If the results were only presented in graphs, these were digitalized and then converted to numbers using the Digitizelt 1.5.7 program (Digitizelt 2003; Bormann, Braunschweig, Germany). Two independent reviewers extracted data from the selected papers, reconciling differences by consensus.

The outcomes measured were as follows: antihistamine effects were assessed by the inhibition rate of histamine-induced wheal and flare (24 h after treatment); safety was assessed by AE frequency, sedative effects frequency and the change of cognitive/psychomotor function scores (3 to 5 h after treatment). Cognitive/psychomotor function scores included critical flicker fusion (CFF), choice reaction time (CRT), compensatory tracking test (CTT), line analogue rating scales for sedation (LARS), and visual analogue score (VAS) of drowsiness, which were used for the assessment of information processing capability, reaction speed, the degree of attention focusing, vigilance and fatigue, somnolence degree. Annotations of cognitive/psychomotor function scores are as follows:

*CFF.* The CFF referred to the frequency of intermittent light stimulation when the flicker happened to achieve fusion and was used to evaluate the information processing ability. Our eyes will produce a sense of flicker when receiving light stimulation with low intermittent frequency. With a gradual increase of the intermittent frequency produced by light stimulation, the flicker gradually disappears. Our eyes will feel a steady and continuous light when the flicker reaches a certain frequency, which is called the fusion of flicker. The decrease in CFF suggested a reduction in the ability to process information.

*CRT.* The CRT was taken as a sensitive measurement of drug-induced changes in psychomotor speed. From a central starting position subjects were required to extinguish one of the six red lights and illuminated at random by touching the appropriate response button. The increase in CRT indicated a reduction in the response speed of subjects.

*CTT.* The CTT was used as a means to assess divided attention. Subjects were required to keep a cursor in alignment with a moving target on a visual display unit screen using a mouse. The evaluation measure of this tracking task was the mean difference between the centers of the target and cursor in pixels, sampled 5 times per second, during the 10 min test period. Higher scores were indicative of less concentration.

*LARS.* The LARS was employed as a measure of the subjective effects of psychoactive drugs. Subjects marked a series of 10 cm line analogue scales, indicating their present feeling with regards to a mid-point, which represented their normal state of mind before treatment began. The mean scores of ratings of ‘tiredness’, ‘drowsiness’, and ‘alertness’, presented among several distracter scales, were taken as a measurement of perceived sedation. The higher the score (in mm), the less alert and more tired and drowsy the subjects felt.

*VAS of drowsiness.* The VAS of drowsiness was used as a subjective indicator of somnolence degree marking by subjects with end points of ‘not drowsy’ and ‘very drowsy’. Higher scores were indicative of more somnolent.

### Risk of Bias assessment

The risk of bias and methodological quality were evaluated using the Cochrane Collaboration tool [8]. There are 6 aspects including (a) sequence generation, (b) allocation concealment, (c) blinding of caregivers, personnel and outcome assessors, (d) incomplete outcome data, (e) selective outcome reporting, and (f) other sources of bias need to be graded as three levels of risk: (A) low risk of bias, (B) unclear risk of bias, and (C) high risk of bias. Two independent reviewers assessed the risk of bias of the selected studies, reconciling differences by consensus.

### Data synthesis

Data analysis was performed using the RevMan 5.3 program (The Cochrane Collaboration, Oxford, UK) and Comprehensive Meta Analysis V2 (Biostat, Englewood, NJ 07631 USA). Pooled weighted mean differences (WMDs) was used for continuous data (the inhibition rate of histamine-induced wheal and flare, the change of cognitive/psychomotor function scores). Odds ratio (OR) was used for dichotomous data (AE frequency, sedative effects frequency). Heterogeneity was assessed by *I*^*2*^ and Cochrane’s Q test. When heterogeneity was not present (*Ι*^2^ < 50%), fixed-effects model (FEM) and Peto OR were applied. For *Ι*^2^ > 50%, a random-effects model (REM) and DerSimonian-Lair OR were used. Potential publication bias was evaluated using funnel plots. Sensitivity analysis was performed by eliminating the selected studies one by one.

## Results

### Search results

**Fig. 1:**
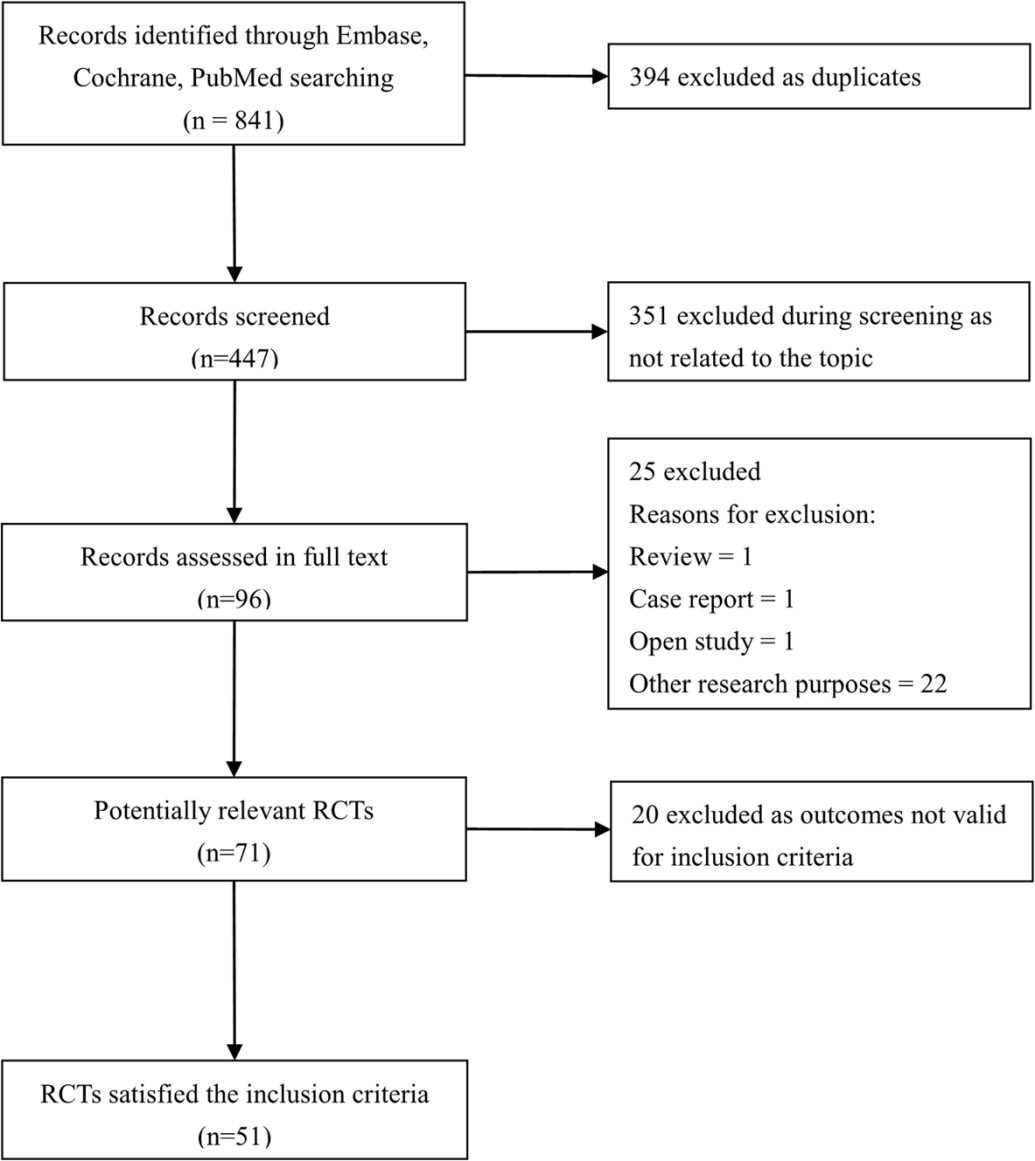
Flow diagram of study selection

As shown in Fig. 1, our search identified 841 records; 394 were excluded due to duplication, 351 were excluded after the first round of screening, and 96 full-text articles were assessed for eligibility. Of these, 25 were excluded because they were reviews, case reports, open studies, or studies aimed at other purposes. Seventy-one clinical trials on the comparison of antihistamine effects or safety of fexofenadine with other antihistamines or placebo for participants were potentially relevant. The second round of screening excluded 20 comparative trials which had outcomes not eligible for inclusion criteria. Finally, 51 RCTs satisfied the inclusion criteria and then were included in our meta-analysis [9–59]. Notably, partial data from 8 RCTs [15, 24, 28, 30, 36, 53, 56, 58] were only reported in graphics and; the attempt to obtain data directly from the authors failed, so graphics were digitized and the SD were estimated using an imputation method.

### Trial characteristics

Of 51 included studies, 20 (39.2%) are parallel and 31 (60.8%) are cross-over trials. The mean age of a total of 14,551 participants included was 31.7 years and 45.0% were male. Among these participants were 3024 cases of healthy subjects, 10,521 cases of AR, 536 cases of CIU, 470 cases of pollinator. Characteristics of the included studies are shown in Table 1.

**Table 1:** Characteristics of included studies

First author, Years	Study design	Subject	Number	Experimental	Comparators	Study duration	Outcome measures
Barbanoj [25], 2003	RCT, crossover study	healthy	18	FEX 120 mg *n* = 18	EBA 20 mg.. *n* = 18; PL *n* = 18	5 days	Wheal, flare, AE, sedative effects
Boyle [36], 2005	RCT, crossover study	healthy	18	FEX 60 mg, *n* = 18	LOR 10 mg, *n* = 18; PL *n* = 18	2 months	Wheal, flare, AE, sedative effects
Larbig [57], 2006	RCT, crossover study	healthy	30	FEX 120 mg, *n* = 30	PL *n* = 30	24 h	Wheal, flare
Simons [55], 2002	RCT, parallel study	healthy	21	FEX 180 mg, *n* = 7	CHL 8 mg, *n* = 7; LOR 10 mg, *n* = 7	9 days	Wheal, flare
Simons [31], 2003	RCT, crossover study	healthy	15	FEX 30 mg, *n* = 15	CET 10 mg, *n* = 15	24 h	Wheal, flare, sedative effects
Takahashi [56], 2004	RCT, crossover study	healthy	7	FEX 120 mg, *n* = 7	BEP 20 mg, *n* = 7; PL *n* = 7	24 h	Wheal, flare, VAS of drowsiness
Finn [11], 1999	RCT, parallel study	CIU	439	FEX 40 mg/ 120 mg/240 mg/480 mg, *n* = 349	PL *n* = 90	4 weeks	AE, sedative effects
Berger [41], 2006	RCT, parallel study	AR	432	FEX 180 mg, *n* = 288	PL *n* = 144	15 days	AE, sedative effects
Berkowitz [42], 2006	RCT, crossover study	AR	63	FEX 180 mg, *n* = 63	PL *n* = 63	2 weeks	AE, sedative effects
Boyle [43], 2006	RCT, crossover study	healthy	18	FEX 120 mg, *n* = 18	CHL 6 mg, n = 18; PL *n* = 18	10 h	AE, sedative effects
Bronsky [10], 1998	RCT, parallel study	AR	548	FEX 80 mg/ 120 mg/ 240 mg, *n* = 411	PL *n* = 137	14 days	AE, sedative effects
Casale [13], 1999	RCT, parallel study	AR	861	FEX 120 mg/ 180 mg, *n* = 569	PL *n* = 292	3 weeks	AE, sedative effects
Van Cauwenberge [17], 2000	RCT, parallel study	AR	685	FEX 120 mg, *n* = 232	LOR 10 mg, *n* = 228; PL *n* = 225	14 days	AE, sedative effects
Day [33], 2004	RCT, parallel study	AR	575	FEX 180 mg, *n* = 239	CET 10 mg, *n* = 240; PL *n* = 96	24 h	AE, sedative effects
Ramesh [50], 2013	RCT, parallel study	AR	50	FEX 120 mg, *n* = 25	CHL 4 mg, *n* = 25	14 days	AE, sedative effects
Grant [14], 1999	RCT, crossover study	healthy	14	FEX 60 mg, *n* = 14	CET 10 mg, *n* = 14; EPI 20 mg, *n* = 14; TER 60 mg, *n* = 14; LOR 10 mg, *n* = 14; PL *n* = 14	24 h	AE, sedative effects
Grant [23], 2002	RCT, crossover study	healthy	18	FEX 180 mg, n = 18	EBA 10 mg, *n* = 18; LOR 10 mg, *n =* 18; MIZ 10 mg, *n* = 18; PL *n* = 18	24 h	AE, sedative effects
Hampel [26], 2003	RCT, parallel study	AR	495	FEX 180 mg, *n* = 248	CET 10 mg, *n* = 247	2 weeks	AE, sedative effects
Hampel [45], 2007	RCT, parallel study	AR	393	FEX 30 mg/ 60 mg, *n* = 193	PL *n* = 200	8 days	AE, sedative effects
Hashiguchi [52], 2016	RCT, crossover study	healthy	126	FEX 120 mg, *n* = 126	BIL 10 mg/ 20 mg, *n* = 252; PL *n* = 126	3 days	AE, sedative effects
Hindmarch [24], 2002	RCT, crossover study	healthy	15	FEX 360 mg, *n* = 15	PRO 30 mg *n* = 15, PL *n* = 15	7 h	AE, sedative effects, CFF, CRT, LARS
Hindmarch [15], 1999	RCT, crossover study	healthy	24	FEX 120 mg, *n* = 24	PRO 30 mg, *n* = 24; LOR 10 mg, *n* = 24; PL *n =* 24	24 h	AE, sedative effects, CFF, CRT, LARS
Horak [20], 2001	RCT, crossover study	AR	40	FEX 120 mg, *n* = 40	CET 10 mg, *n* = 40; PL *n* = 40	2 days	AE, sedative effects
Horak [48], 2010	RCT, crossover study	allergic volunteers	75	FEX 120 mg, *n* = 70	CET 10 mg, *n* = 68; BIL 20 mg, *n* = 74; PL *n* = 70	2 days	AE, sedative effects
Horak [34], 2005	RCT, crossover study	allergic volunteers	94	FEX 120 mg, *n* = 94	PL *n* = 94	2 days	AE, sedative effects
Howarth [16], 1999	RCT, parallel study	AR	722	FEX 120 mg/ 180 mg, *n* = 421	CET 10 mg, *n* = 209; PL *n* = 209	2 weeks	AE, sedative effects
Inami [53], 2016	RCT, crossover study	healthy	20	FEX 60 mg, *n* = 20	DIP 50 mg, *n =* 20; PL *n* = 20	6 h	AE, sedative effects, LARS
Kaiser [47], 2008	RCT, parallel study	AR	835	FEX 120 mg, *n* = 359	LOR 10 mg, *n* = 357; PL *n* = 119	7 days	AE, sedative effects
Kaiser [21], 2001	RCT, parallel study	AR	836	FEX 120 mg, *n* = 360	LOR 10 mg, *n* = 357; PL *n* = 119	7 days	AE, sedative effects
Kamei [49], 2012	RCT, crossover study	healthy	24	FEX 60 mg, *n* = 24	PRO 25 mg, *n* = 24; OLO 5 mg, *n* = 24; PL *n* = 24	8 h	AE, sedative effects, CFF, CRT, LARS
Kamei [28], 2003	RCT, crossover study	healthy	11	FEX 120 mg, *n* = 11	d-CHL 4 mg, *n* = 11; OLO 10 mg, *n* = 11; PL *n* = 11	8 h	AE, sedative effects, CFF, CRT, CTT, LARS
Mansfield [29], 2003	RCT, crossover study	healthy	42	FEX 180 mg, *n* = 42	DIP 50 mg, *n* = 42; PL *n* = 42	2 h	AE, sedative effects
Milgrom [46], 2007	RCT, parallel study	AR	453	FEX 60 mg, *n* = 222	PL *n* = 231	2 weeks	AE, sedative effects
Okubo [44], 2006	RCT, crossover study	healthy	9	FEX 60 mg, *n* = 9	EPI 20 mg, *n* = 9; PL *n* = 9	5 h	AE, sedative effects
Okubo [54], 2016	RCT, parallel study	AR	747	FEX 120 mg, *n* = 247	BIL 20 mg, *n* = 249; PL *n* = 251	2 weeks	AE, sedative effects
Prenner [18], 2000	RCT, crossover study	AR	929	FEX 120 mg, *n* = 457	LOR 10 mg, *n* = 472	14 days	AE, sedative effects
Purohit [22], 2001	RCT, crossover study	healthy	26	FEX 120 mg/ 180 mg, *n* = 52	CET 10 mg, *n* = 26; PL *n* = 26	24 h	AE, sedative effects
Purohit [35], 2004	RCT, crossover study	healthy	42	FEX 180 mg, n = 42	CET 10 mg, *n* = 42	4 h	AE, sedative effects
Ridout [30], 2003	RCT, crossover study	healthy	18	FEX 180 mg, *n* = 18	HYD 50 mg, *n* = 18; PL *n =* 18	5 h	AE, sedative effects, CFF, CRT, LARS
Schapowal [39], 2005	RCT, parallel study	AR	220	FEX 180 mg, *n* = 113	PL *n* = 107	14 days	AE, sedative effects
Schoepke [51], 2013	RCT, crossover study	healthy	18	FEX 120 mg, *n* = 18	PL *n* = 18	24 h	AE, sedative effects
Simons [9], 1997	RCT, crossover study	healthy	20	FEX 120 mg, *n* = 40	LOR 10 mg, *n* = 20 PL *n* = 20	24 h	AE, sedative effects
Tsuda [40], 2005	RCT, crossover study	healthy	10	FEX 120 mg, *n* = 10	CET 5 mg/10 mg, *n* = 20; LOR 10 mg, *n* = 10; PL *n* = 10	24 h	AE, sedative effects
Wahn [32], 2003	RCT, parallel study	AR	935	FEX 30 mg, *n* = 464	PL *n* = 471	14 days	AE, sedative effects
Weiler [19], 2000	RCT, crossover study	healthy	40	FEX 60 mg, *n* = 40	DIP 50 mg, *n* = 40; PL *n* = 40	5 h	AE, sedative effects, VAS of drowsiness
Ballmer-Weber [12], 1999	RCT, crossover study	healthy	20	FEX 180 mg, *n* = 20	CET 10 mg, *n* = 40; ACR 8 mg, *n* = 20	1 h	sedative effects
Day [37], 2005	RCT, parallel study	AR	599	FEX 180 mg, *n* = 250	CET 10 mg, *n* = 249; PL *n* = 100	7 h	sedative effects
Handa [27], 2004	RCT, parallel study	CIU	97	FEX 180 mg, *n* = 45	CET 10 mg, *n* = 52	28 days	sedative effects
Hyo [38], 2005	RCT, parallel study	healthy	113	FEX 120 mg, *n* = 28	CET 10 mg, *n* = 30; LOR 10 mg, *n* = 28; PL *n* = 27	2 days	sedative effects
Ridout [58], 2002	RCT, crossover study	healthy	24	FEX 120 mg, *n* = 24	PRO 25 mg, *n* = 24; PL *n* = 24	8 h	CFF, CRT, CTT, LARS
Naicker [59], 2013	RCT, crossover study	healthy	11	FEX 180 mg, *n* = 11	PRO 25 mg, *n* = 11; LOR 10 mg, *n* = 11; PL *n* = 11	3 h	CRT, CTT, VAS of drowsiness

### Antihistamine effects

#### The inhibition rate of histamine-induced wheal

**Fig. 2:**
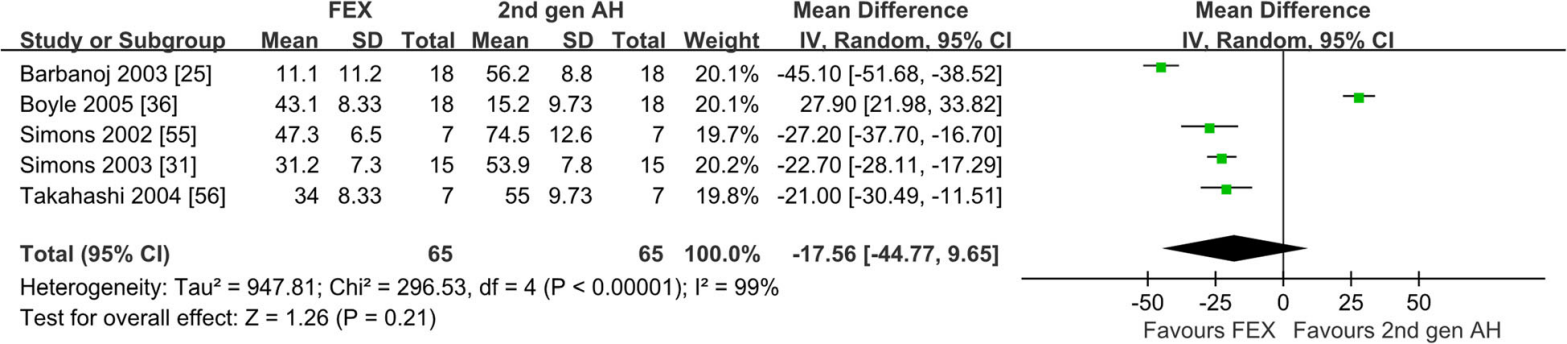
Suppression Percentage of histamines-induced wheal: fexofenadine vs. the second-generation antihistamines. *2nd gen AH* second generation antihistamines

Six studies reported the inhibition rate of histamine-induced wheal after taking fexofenadine [25, 31, 36, 55–57]. Of the 6 studies on healthy subjects, 1 compared with the first-generation antihistamines [55], 5 compared with the second-generation antihistamines [25, 31, 36, 55, 56], and 5 compared with placebo [25, 31, 36, 56, 57]. The comparison between fexofenadine and the first-generation antihistamines was not pooled for meta-analysis because there was only 1 study included. When compared with the second-generation antihistamines, as shown in Fig. 2, the inhibition rates of histamine-induced wheal were not different (WMD = − 17.56; 95% CI: − 44.77 to 9.65, *P* = 0.21). The heterogeneity was 99%, which may be generated from the inconsistent doses of fexofenadine and types of the second-generation antihistamines. When compared with placebo, the results indicated that fexofenadine produced significantly higher inhibition rate of histamine-induced wheal. After sensitivity analysis and checking the trial methods, 2 studies were excluded because of different study duration compared with other studies [25, 36]. As shown in Additional file 2: Figure S2 the inhibition rate of histamine-induced wheal of fexofenadine was significantly higher than that of placebo (WMD = -18.93; 95% CI: 15.29 to 22.57, *P* < 0.00001). A *Ι*^2^ of 8% represents low heterogeneity.

#### The inhibition rate of histamine-induced flare

**Fig. 3:**
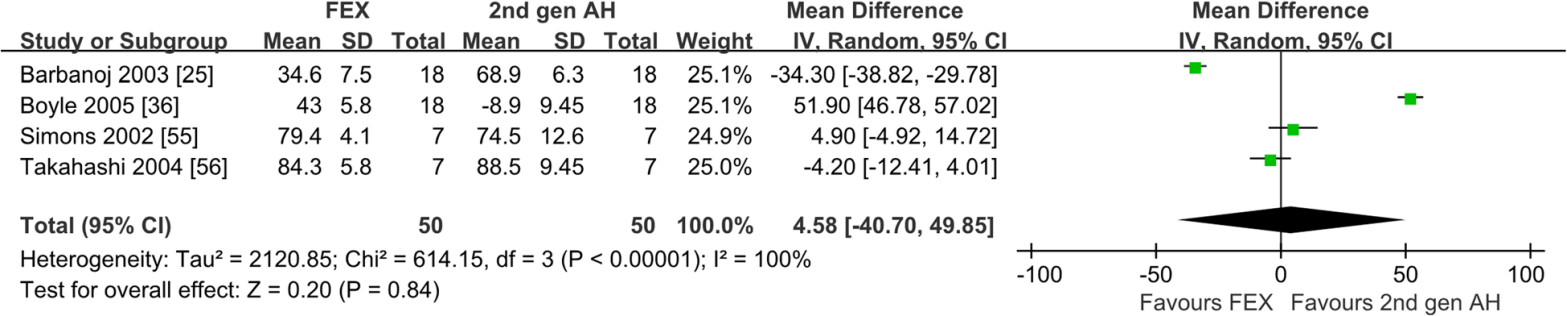
Suppression Percentage of histamines-induced flare: fexofenadine vs. the second-generation antihistamines. *2nd gen AH* second generation antihistamines

Six studies reported the inhibition rate of histamine-induced flare after taking fexofenadine [25, 31, 36, 55–57]. Of the 6 studies on healthy subjects, only 1 compared with the first-generation antihistamines [55], which was not suitable for meta-analysis. Four studies compared with the second-generation antihistamines [25, 36, 55, 56], as shown in Fig. 3, the inhibition rate of histamine-induced flare were not different (WMD = 4.58; 95% CI − 40.70 to 49.85, *P* = 0.84). Five studies compared with placebo [25, 31, 36, 56, 57], as shown in Additional file 3: Figure S3, fexofenadine produced significantly higher inhibition rate of histamine-induced flare (WMD = 35.75, 95% CI: 18.67 to 52.83, *P* < 0.00001). The heterogeneity may be generated from the inconsistent doses of fexofenadine and different type of the second-generation antihistamines. Sensitivity analysis showed the meta-analysis results were similar.

### Safety

#### Adverse events frequency

**Fig. 4:**
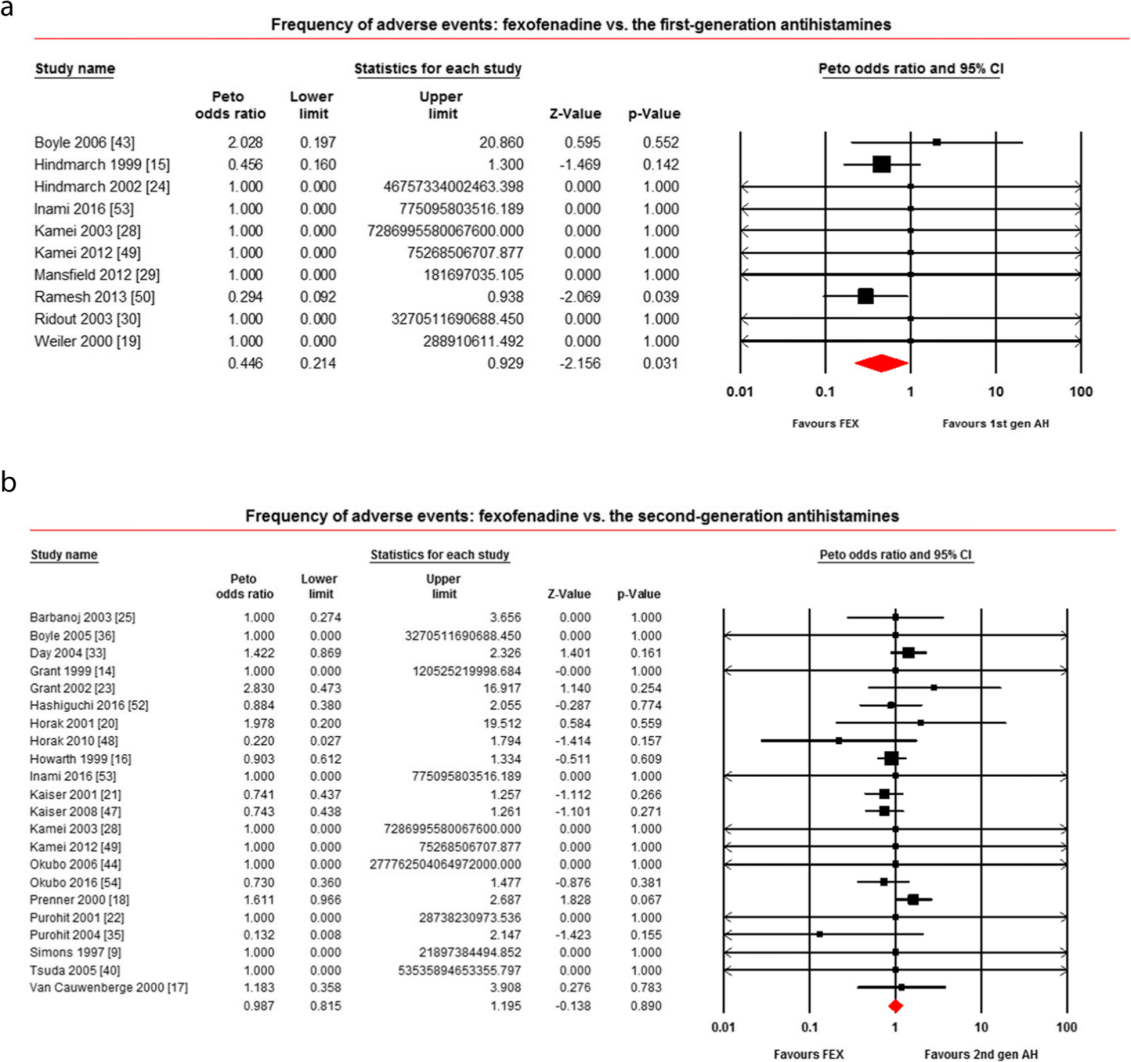
Frequency of adverse events: **a** fexofenadine vs. the first-generation antihistamines; **b** fexofenadine vs. the second-generation antihistamines. *1st gen AH* the first-generation antihistamines, *2nd gen AH* second generation antihistamines

Forty-one studies reported AE after taking fexofenadine [9–11, 13–26, 28–30, 32–36, 39–54]. Of 41 studies, 10 compared with the first-generation antihistamines [15, 19, 24, 28–30, 43, 49, 50, 53], 22 compared with the second-generation antihistamines [9, 14, 16–18, 20–23, 25, 28, 33, 35, 36, 40, 44, 47–49, 52–54], and 37 compared with placebo [9–11, 13–17, 19–25, 28–30, 32–34, 36, 39–49, 51–54]. When compared with the first-generation antihistamines, as demonstrated in Fig. 4a, fexofenadine produced significantly lower AE frequency (OR = 0.446; 95% CI: 0.214 to 0.929, *P* = 0.031). When compared with the second-generation antihistamines, as shown in Fig. 4b, the AE frequency for fexofenadine versus the second-generation antihistamines were not different (OR = 0.987; 95% CI: 0.815 to 1.195, *P* = 0.890). When compared with placebo, as shown in Additional file 4: Figure S4, the AE frequency of these two groups were not different (OR = 0.999; 95% CI: 0.863 to 1.156, *P* = 0.987).

#### Sedative effects frequency

**Fig. 5:**
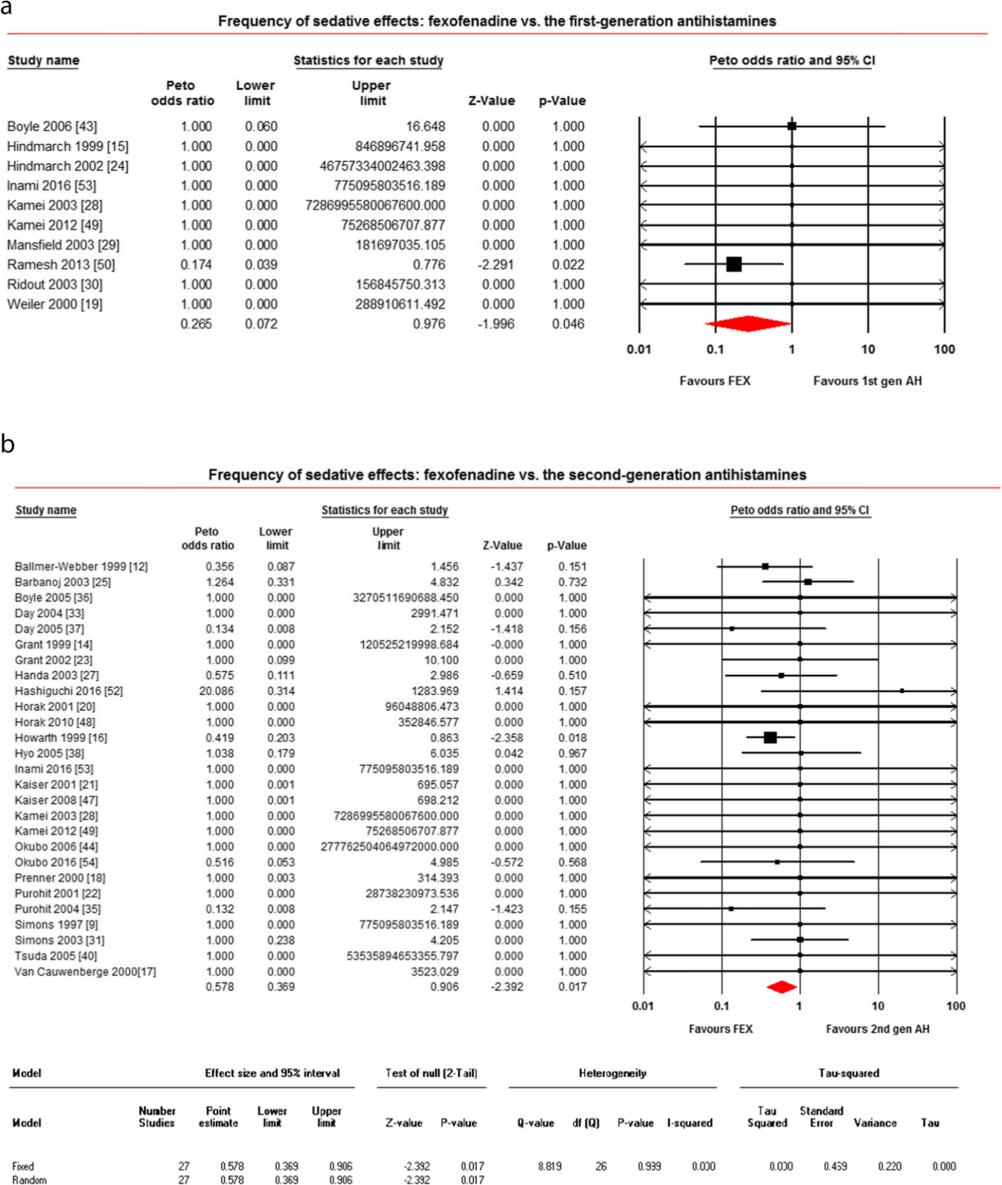
Frequency of sedative effects: **a** fexofenadine vs. the first-generation antihistamines; **b** fexofenadine vs. the second-generation antihistamines. *1st gen AH* the first-generation antihistamines, *2nd gen AH* second generation antihistamines

Forty-six studies reported sedative effects frequency after taking fexofenadine [9–54]. Of 46 studies, 10 compared with the first-generation antihistamines [15, 19, 24, 28–30, 43, 49, 50, 53], 27 compared with the second-generation antihistamines [9, 12, 14, 16–18, 20–23, 25, 27, 28, 31, 33, 35–38, 40, 44, 47–49, 52–54], and 38 compared with placebo [9–11, 13–17, 19–25, 28–30, 32–34, 36, 38–49, 51–54]. When compared with the first-generation antihistamines, as shown in Fig. 5a, fexofenadine produced significantly lower sedative effects frequency (OR = 0.265; 95% CI: 0.072 to 0.976, *P* = 0.046). When compared with the second-generation antihistamines, as shown in Fig. 5b, fexofenadine produced significantly lower sedative effects frequency (OR = 0.578; 95% CI: 0.369 to 0.906, *P* = 0.017). When compared with placebo, as shown in Additional file 5: Figure S5, the sedative effects frequency for fexofenadine versus placebo were not different (OR = 1.608; 95% CI: 0.884 to 2.924, *P* = 0.120), but not statistically significant (OR 1.6 [0.8–2.9]). Five studies (18, 432, 126, 113, 747 patients respectively) showed more AE for fexofenadine than placebo and none of the others showed that for placebo more AEs than fexofenadine.

#### Cognitive/psychomotor function

**Fig. 6:**
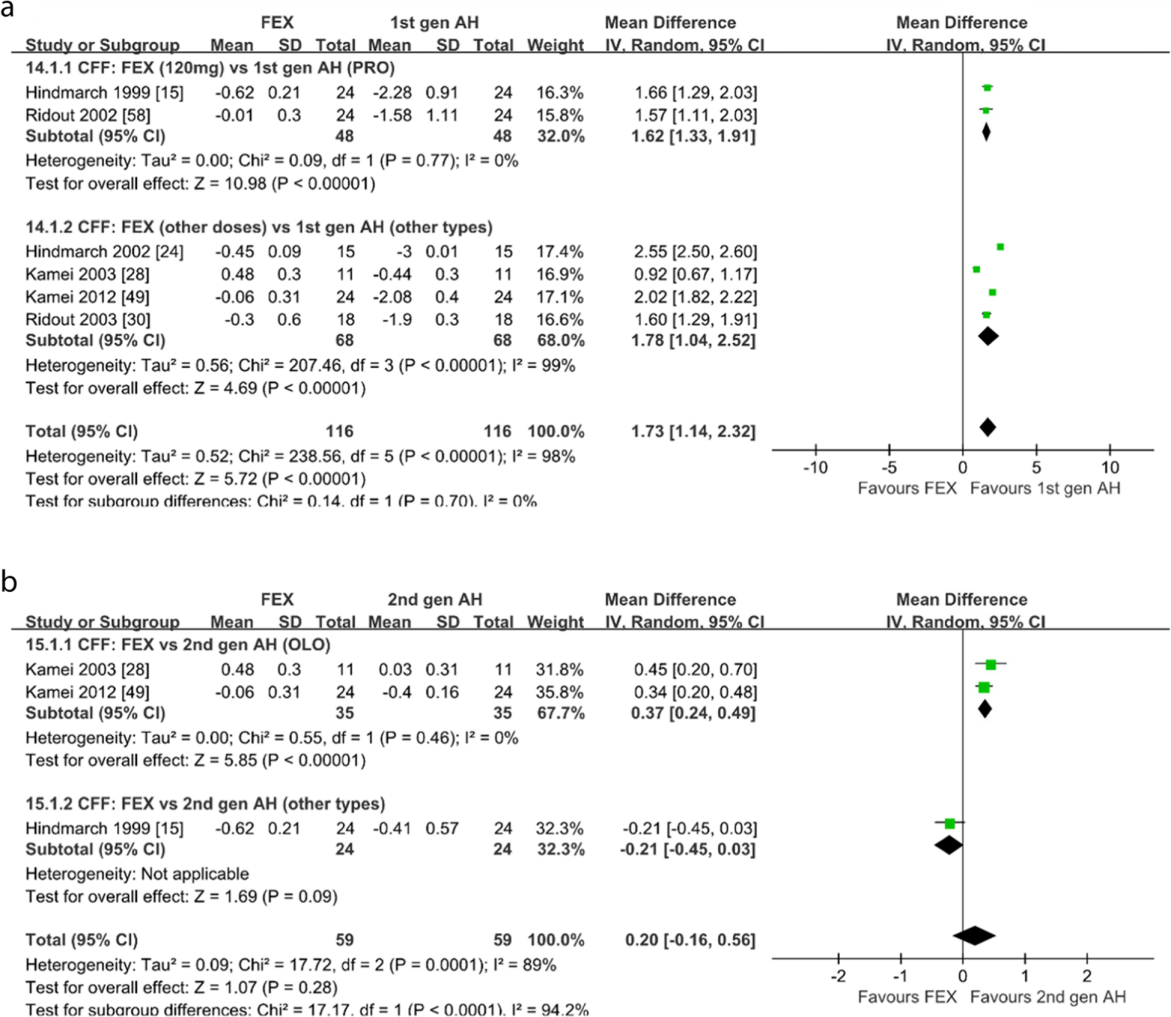
Critical flicker fusion: **a** fexofenadine vs. the first-generation antihistamines; **b** fexofenadine vs. the second-generation antihistamines. *1st gen AH* the first-generation antihistamines, *2nd gen AH* second generation antihistamines

*CFF.* Six studies reported the CFF of subjects after taking fexofenadine [15, 24, 28, 30, 49, 58]. Of the 6 studies on healthy subjects, 3 compared with the second-generation antihistamines [15, 28, 49], 6 compared with the first-generation antihistamines and placebo [15, 24, 28, 30, 49, 58]. When compared with the first-generation antihistamines, as shown in Fig. 6a, fexofenadine produced significantly less change of CFF (WMD = 1.73; 95% CI: 1.14 to 2.32, *P* < 0.00001) and the subgroup-analysis showed that fexofenadine 120 mg/d produced significantly less change of CFF compared with promethazine (WMD = 1.62; 95% CI: 1.33 to 1.91, *P* < 0.00001). When compared with the second-generation antihistamines, as shown in Fig. 6b, the change of CFF were not different (WMD = 0.20; 95% CI: − 0.16 to 0.56, *P* = 0.28) and the subgroup-analysis showed that fexofenadine produced significantly less change of CFF compared with olopatadine (WMD = 0.37; 95% CI: 0.24 to 0.49, *P* < 0.00001). When compared with placebo, as shown in Additional file 6: Figure S6, the change of CFF were not different (WMD = − 0.15; 95% CI: − 0.37 to 0.06, *P* = 0.17). After checking the trial methods, we found that the heterogeneity for fexofenadine versus placebo may be generated from the inconsistent doses of fexofenadine. Sensitivity analysis showed the meta-analysis results were similar.

**Fig. 7:**
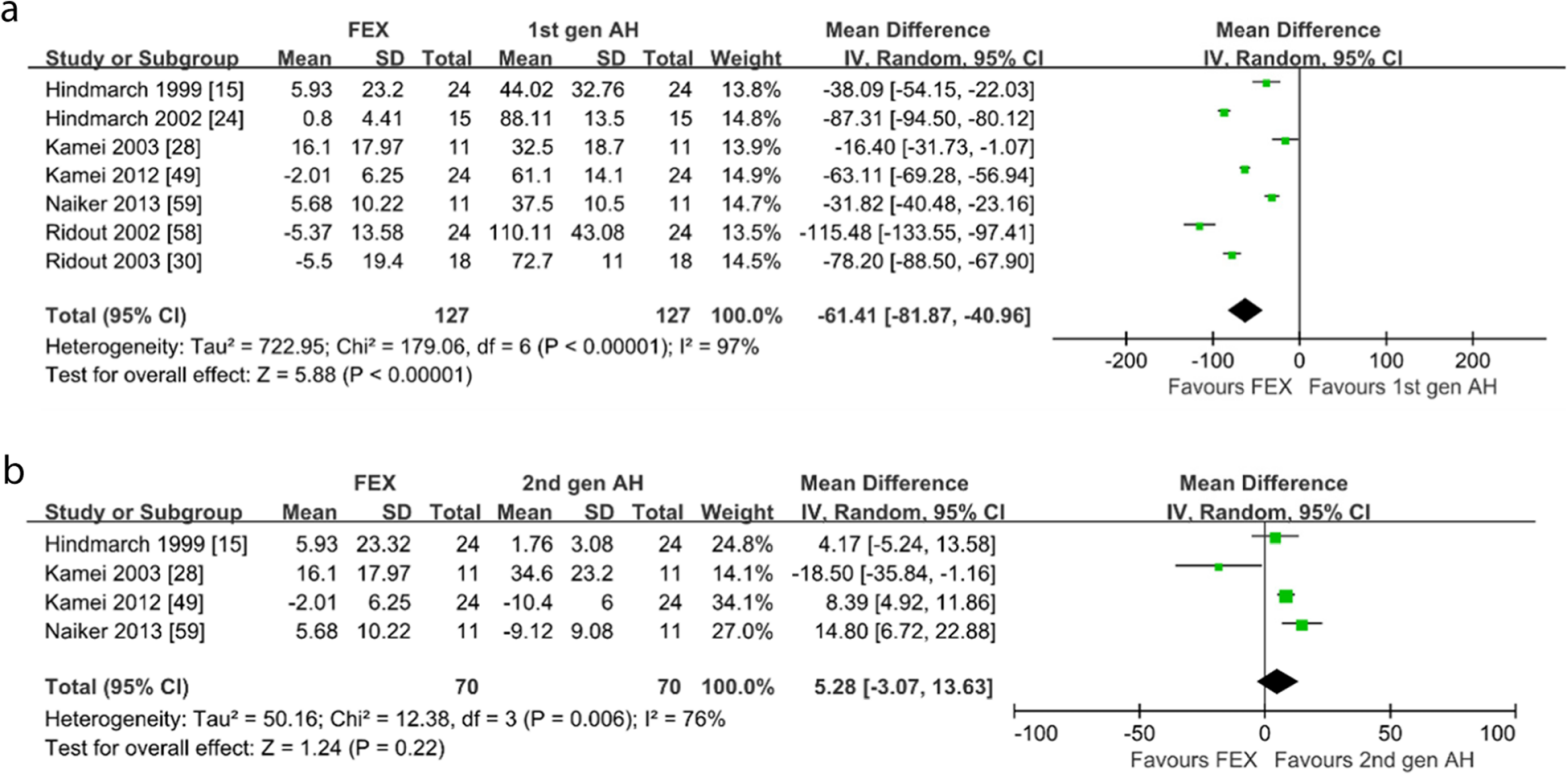
Choice reaction time: **a** fexofenadine vs. the first-generation antihistamines; **b** fexofenadine vs. the second-generation antihistamines. *1st gen AH* the first-generation antihistamines, *2nd gen AH* second generation antihistamines

*CRT.* Seven studies reported the CRT of subjects after taking fexofenadine [15, 24, 28, 30, 49, 58, 59]. Of the 7 studies on healthy subjects, all of the included studies compared with the first-generation antihistamines [15, 24, 28, 30, 49, 58, 59], as shown in Fig. 7a, fexofenadine produced significantly less change of CRT (WMD = − 61.41; 95% CI: − 81.87 to − 40.96, *P* < 0.00001). Four studies compared with the second-generation antihistamines [15, 28, 49, 59], as shown in Fig. 7b, the change of CRT were not different (WMD = 5.28; 95% CI: − 3.07 to 13.63, *P* = 0.22). Six studies compared with placebo [24, 28, 30, 49, 58, 59], as shown in Additional file 7: Figure S7, the change of CRT were not different (WMD = 3.68; 95% CI: − 2.95 to 10.32, *P* = 0.28). The heterogeneity may be generated from the inconsistent doses of fexofenadine and different antihistamines in each comparator. Sensitivity analysis showed the meta-analysis results were similar.

**Fig. 8:**
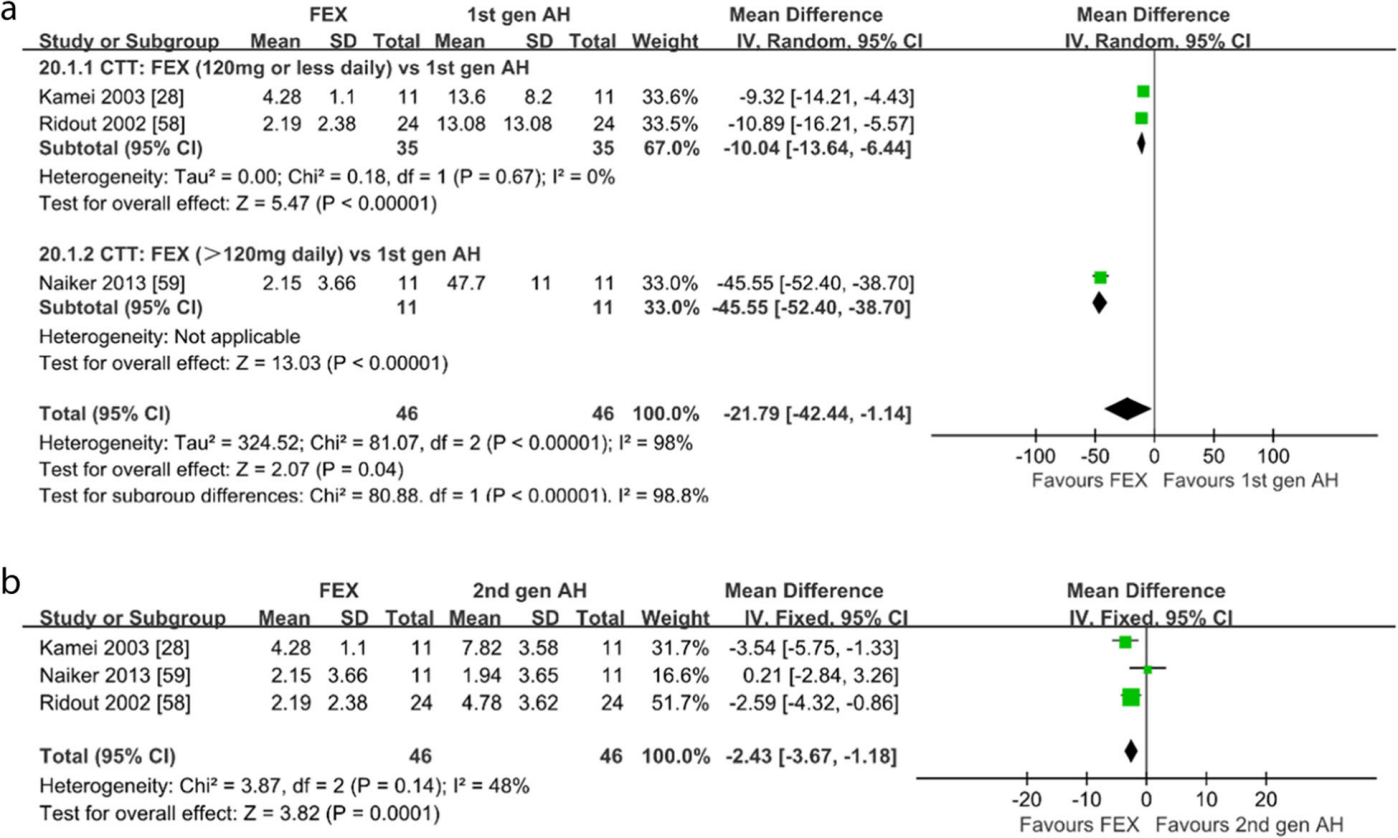
Compensatory tracking task: **a** fexofenadine vs. the first-generation antihistamines; **b** fexofenadine vs. the second-generation antihistamines. *1st gen AH* the first-generation antihistamines, *2nd gen AH* second generation antihistamines

*CTT.* Three studies on healthy subjects compared the CTT of subjects with the first-generation antihistamines, the second-generation antihistamines and placebo [28, 58, 59]. When compared with the first-generation antihistamines, as shown in Fig. 8a, fexofenadine produced significantly less change of CTT (WMD = − 21.79; 95% CI: − 42.44 to − 1.14, *P* = 0.04) and the subgroup-analysis showed that fexofenadine 120 mg/d or less dose produced significantly less change of CTT (WMD = − 10.04; 95% CI: − 13.16 to − 6.44, *P* < 0.00001). When compared with the second-generation antihistamines, as shown in Fig. 8b, fexofenadine produced significantly less change of CTT (WMD = − 2.43; 95% CI: − 3.67 to − 1.18, *P* = 0.0001). When compared with placebo, as shown in Additional file 8: Figure S8, the change of CTT were not different (WMD = 0.11; 95% CI: − 3.81 to 4.02, *P* = 0.96). Sensitivity analysis showed the meta-analysis results were similar.

**Fig. 9:**
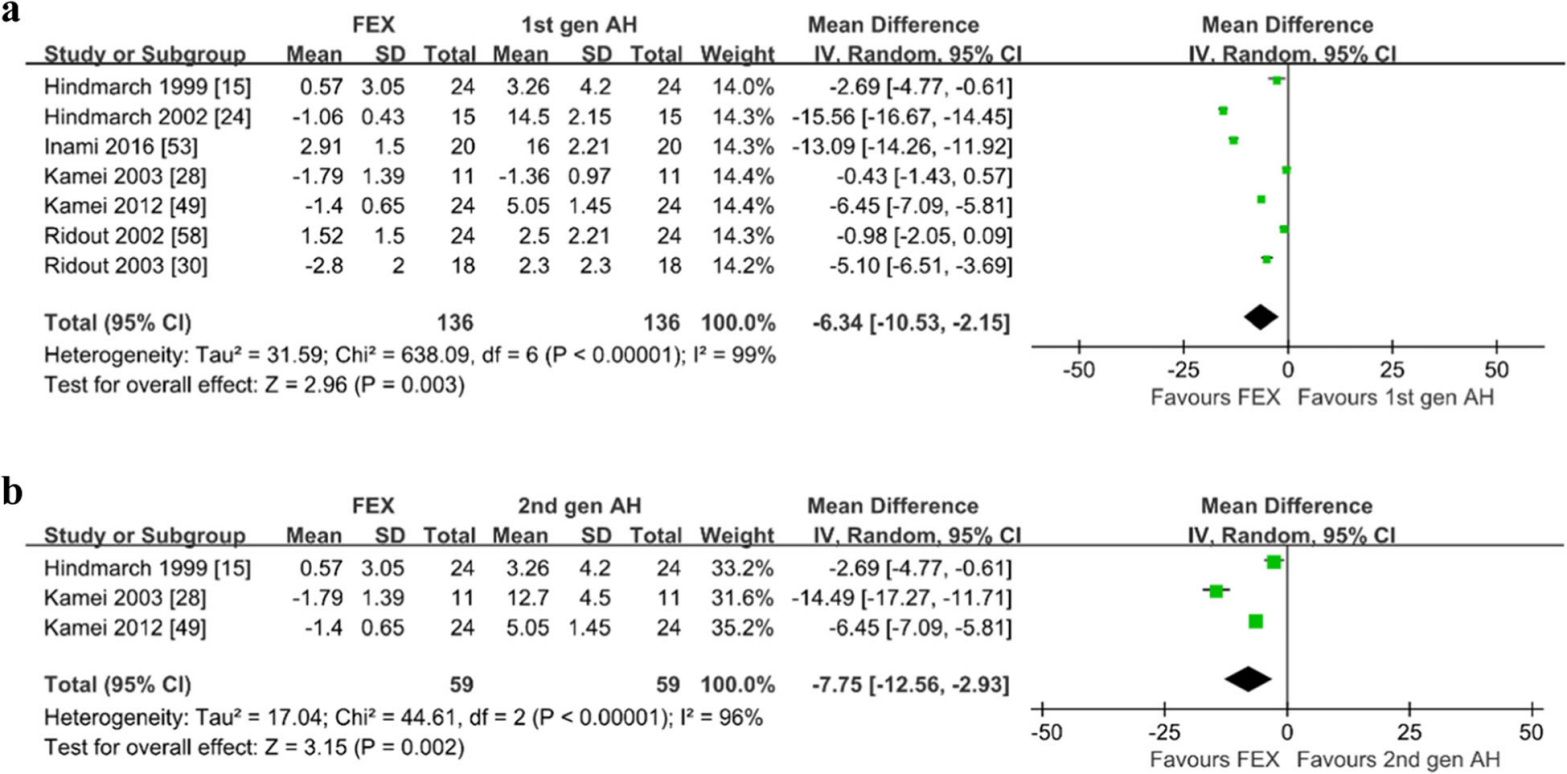
Line analogue rating scales for sedation: **a** fexofenadine vs. the first-generation antihistamines; **b** fexofenadine vs. the second-generation antihistamines. *1st gen AH* the first-generation antihistamines, *2nd gen AH* second generation antihistamines

*LARS.* Seven studies reported the LARS of subjects after taking fexofenadine [15, 24, 28, 30, 49, 53, 58]. Of the 7 studies on healthy subjects, 3 compared with the second-generation antihistamines [15, 28, 49], all compared with the first-generation antihistamines and placebo [15, 24, 28, 30, 49, 53, 58]. As shown in Fig. 9 and Additional file 9: Figure S9, fexofenadine produced significantly less change of LARS when compared with the first-generation antihistamines (WMD = − 6.34; 95% CI: − 10.53 to − 2.15, *P* = 0.003), the second-generation antihistamines (WMD = − 7.75; 95% CI: − 12.56 to − 2.93, *P* = 0.002) and placebo (WMD = − 2.67; 95% CI: − 3.99 to − 1.35, *P* < 0.0001). The heterogeneity may be generated from the inconsistent doses of fexofenadine and different antihistamines in each comparator. Sensitivity analysis showed the meta-analysis results were similar.

**Fig. 10:**
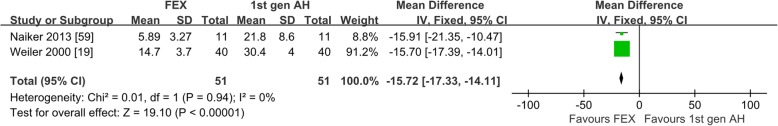
Visual analogue drowsiness scores: fexofenadine vs. the first-generation antihistamines. *1st gen AH* the first-generation antihistamines

*VAS of drowsiness.* Three studies reported the VAS of drowsiness of subjects after taking fexofenadine [19, 56, 59]. Of the 3 studies on healthy subjects, 2 compared with the first-generation antihistamines [19, 59], 2 compared with the second-generation antihistamines [56, 59], all compared with placebo [19, 56, 59]. The comparison between fexofenadine and the second-generation antihistamines was not pooled for meta-analysis because there was only 2 study included with a high heterogeneity. When compared with the first-generation antihistamines, as shown in Fig. 10, fexofenadine produced significantly less change of VAS of drowsiness (WMD = − 15.72; 95% CI: − 17.33 to − 14.11, *P* < 0.00001). When compared with placebo, as shown in Additional file 10: Figure S10, the change of VAS of drowsiness were not different (WMD = 7.18; 95% CI: − 0.64 to 14.99, *P* = 0.07). The heterogeneity may be generated from the inconsistent doses of fexofenadine. Sensitivity analysis showed the meta-analysis results were similar. Results summary is shown in Table 2.

**Table 2:** Results summary

Outcome measures		FEX vs. 1st gen AH				FEX vs. 2nd gen AH				FEX vs. PL			
		Included studies	number	Heterogeneity (%)	*P*	Included studies	number	Heterogeneity (%)	*P*	Included studies	number	Heterogeneity (%)	*P*
Antihistamine effects	Inhibition rate of wheal					5	130	99	0.210	5	104	8	<0.001
	Inhibition rate of flare					4	100	100	0.840	5	176	96	< 0.001
Safety profiles	AE	10	522	0	0.031	22	5699	0	0.890	37	8591	0	0.987
	SE	10	522	0	0.046	27	6471	0	0.002	38	8700	0	0.120
	CFF	6	232	98	<0.001	3	118	89	0.280	6	232	85	0.170
	CRT	7	254	97	<0.001	4	140	76	0.220	6	206	89	0.280
	CTT	3	92	98	0.040	3	92	48	<0.001	3	92	95	0.960
	LARS	7	272	99	0.003	3	118	96	0.002	7	272	90	<0.001
	VAS of drowsiness	2	102	0	<0.001					3	116	97	0.070

### Risk of Bias

As shown in Additional file 1: Figure S1, most (82%) of included studies had low risk of bias in random sequence generation. Twenty-two percent had low risk of bias in allocation concealment. Twenty-seven percent had low risk of bias in blinding of outcome assessment. All studies had low risk of bias in incomplete outcome data and selective reporting.

### Publication Bias

Symmetry was shown in funnel plots when the safety of fexofenadine was compared to other antihistamines and placebo (Additional files 11: Figures S11, Additional file 12: Figure S12, Additional file 13: Figure S13, Additional file 14: Figure S14, Additional file 15: Figure S15, Additional file 16: Figure S16, Additional file 17: Figure S17, Additional file 18: Figure S18, Additional file 19: Figure S19), which means no publication bias in these analyses. A modest asymmetric funnel was shown when the antihistamine effect of fexofenadine was compared to other antihistamines and placebo (Additional files 11: Figure S11, Additional file 12: Figure S12, Additional file 13: Figure S13, Additional file 14: Figure S14, Additional file 15: Figure S15, Additional file 16: Figure S16, Additional file 17: Figure S17, Additional file 18: Figure S18, Additional file 19: Figure S19), which means no significant publication bias in these analyses.

## Discussion

Based on our review of the literature, this is the first meta-analysis to assess the antihistamine effects and sedative effects of fexofenadine. Our meta-analysis indicates that fexofenadine has better safety profiles compared to the second-generation antihistamines. The antihistamine effects (the inhibition rate of histamine-induced wheal and flare) of fexofenadine were significantly higher than that of placebo and were not significantly different compared with the second-generation antihistamines.

Antihistamine effects that assessed by the inhibition rate of histamine-induced wheal and flare are important measurements to evaluate the efficacy of antihistamines in the treatment of allergic diseases [60]. Based on the pooled analysis, we find that the antihistamine effects and duration of fexofenadine are probably no worse than that of the second-generation antihistamines and more positive than that of placebo. Similarly to our study, a recent systematic review and meta-analysis showed that fexofenadine was effective on the treatment of nasal symptoms in patients with seasonal allergic rhinitis (SAR) [61]. On the contrast to that systematic review, our study includes all RCTs involving fexofenadine treatment that compared with other antihistamines or placebo by evaluating the inhibition rate of histamine-induced wheal and flare, not only just SAR RCTs. Therefore, fexofenadine that has positive antihistamine effects is suitable for most of the patients with indications requiring antihistamines. Of note, a study suggested that the inhibition rate of fexofenadine on histamine-induced wheal was lower than that of loratadine [36], while another study showed that fexofenadine had a significantly higher inhibition rate on histamine-induced wheal compared with loratadine [55]. The reason for this may be the different doses of fexofenadine (60 mg/d and 180 mg/d) and the same dose of loratadine (10 mg/d) in the two study. Therefore, further studies are required to explore a more secure and effective dose of fexofenadine that compared with loratadine.

AE is closely related to drug damage to the body [62]. AE in included studies are as follows: headache, drowsiness, fatigue, upper respiratory infection, asthma, pharyngitis, dry mouth, cough, nausea, gastrointestinal pain, diarrhea, rash, epistaxis, sinusitis, back pain, leukopenia, etc. In the above AE, headache is the most common AE in subjects who treated with fexofenadine. Overall, fexofenadine is well-tolerated and discontinuation owing to side effects generally occurs in < 5% of patients [63]. All the first-generation antihistamines and most of the second-generation antihistamines cause cardiotoxicity by inhibiting muscarinic cholinoreceptor (M-ChR), which can regulate heart rate, heart rhythm and cardiac muscle [64]. A previous study indicated that fexofenadine did not prolong QT interval and cause arrhythmia when it was used alone or combined with other drugs such as ketoconazole and erythromycin [65]. A dog model showed that fexofenadine was 600 times more affinity for H1 receptor than M-ChR, while desloratadine was only 5 times than M-ChR although it was also a new generation antihistamine, indicating that fexofenadine may have no cardiotoxicity [66]. This study find that there is no hepatotoxicity or cardiotoxicity related AE in subjects treated with fexofenadine, further supporting that fexofenadine may have no hepatotoxicity and cardiotoxicity. A systematic review and meta-analysis showed that there was no significant difference of AE frequency between fexofenadine and placebo in patients with SAR [61]. Consistently in the respect of AE, our study indicates that the safety profile of fexofenadine is more positive than that of the first-generation antihistamines and similar to the second-generation antihistamines and placebo.

Sedative effect is one of the most concerned issue of AE [67]. The current study indicates that the risk of fexofenadine on sedative effects is lower than that of the first-generation antihistamines and the second-generation antihistamines, and similar to placebo. A previous study showed that fexofenadine may have no sedative effect or only have mild sedative effects since fexofenadine could not pass the blood-brain barrier [68]. Our study demonstrates that the risk of fexofenadine on sedative effects was lower than that of the first-generation antihistamines and the second-generation antihistamines and even has been as low as placebo. A recent meta-analysis showed that levocetirizine had a mild sedative effects although it was a new generation antihistamine [69]. According to our study, fexofenadine with no sedative effect is more worthy of recommendation among the new generation antihistamines. Based on the fact that fexofenadine may have no sedative effect, an expert consensus in the United States recommended National Aeronautics and Space Administration (NASA) to authorize pilots to use fexofenadine if necessary [70].

The cognitive/psychomotor function is another important relevant issue of AE [71]. This study suggests that fexofenadine has less cognitive/psychomotor impairment compared with both of the first-generation antihistamines and the second-generation antihistamines. In addition, the cognitive/psychomotor impairment of fexofenadine is similar to placebo. A recent systematic review suggested that fexofenadine was ranked as the least psychomotor impairment antihistamines compared with all other antihistamines in Japanese market [72]. In contrast to that systematic review, our study included antihistamines used worldwide. Furthermore, we added a comparison between fexofenadine and placebo. The results further identify the fact that fexofenadine may have no cognitive/psychomotor impairment. As for CFF, we find that fexofenadine has a positive information processing capability compared with promethazine. Similarly, a study showed that the information processing capability after treating with fexofenadine 120 mg/d or 60 mg/d was better than olopatadine 10 mg/d or 5 mg/d [28, 49]. But another study demonstrated that the information processing capability after treating with fexofenadine 120 mg/d was worse than that of loratadine 10 mg/d [15]. The above discordance may be attributed to the differences of washout period. Only 4 days applied in study of Hindmarch may result in insufficient drug clearance, which affected the reliability of its result. Therefore, although fexofenadine has marginal cognitive/psychomotor impairment compared with the second-generation antihistamines, the comparison on cognitive/psychomotor function between fexofenadine and individual second-generation antihistamine remains to be further explored.

There are several potential limitations in this study. The setting of subgroups regarding dose/duration/type of antihistamines was unavailable because of limited studies. The comparison of antihistamine effects and cognitive/psychomotor function were lack of large sample RCTs, which may increase the risk of bias. The way to obtain outcome measures such as the frequency of AE and sedative effects was different.

## Conclusions

Fexofenadine has a positive antihistamine effect, which is probably no worse than the second-generation antihistamines. Fexofenadine probably has a favorable safety profile, which is more likely better than that of the first-generation antihistamines. There is lack of data to support that fexofenadine has a better overall safety profile compared to the second-generation antihistamines, however, some presently available evidence on sedative effects and certain aspects of cognitive/psychomotor function favors fexofenadine. Therefore, fexofenadine may be worthy of recommendation for safety related workers. However, more multicenter, large sample, long-term follow-up and well-designed head-to-head trials are required to the further understanding of the efficacy and safety of fexofenadine.

## Supplementary information

**Additional file 1: Figure S1.** Risk of bias: a risk of bias summary; b risk of bias graph.

**Additional file 2.** **Figure S2.** Forest plot of wheal for FEX vs. PL.

**Additional file 3.** **Figure S3.** Forest plot of flare for FEX vs. PL.

**Additional file 4.** **Figure S4.** Forest plot of AE for FEX vs. PL.

**Additional file 5.** **Figure S5.** Forest plot of SE for FEX vs. PL.

**Additional file 6.** **Figure S6.** Forest plot of CFF for FEX vs. PL.

**Additional file 7.** **Figure S7.** Forest plot of CRT for FEX vs. PL.

**Additional file 8.** **Figure S8.** Forest plot of CTT for FEX vs. PL.

**Additional file 9.** **Figure S9.** Forest plot of LARS for FEX vs. PL.

**Additional file 10.** **Figure S10.** Forest plot of VAS of drowsiness for FEX vs. PL.

**Additional file 11: Figure S11.** Funnel plot of wheal: a fexofenadine vs. the second-generation antihistamines; b fexofenadine vs. placebo.

**Additional file 12: Figure S12.** Funnel plot of flare: a fexofenadine vs. the second-generation antihistamines; b fexofenadine vs. placebo.

**Additional file 13: Figure S13.** Funnel plot of adverse events: a fexofenadine vs. the first-generation antihistamines; b fexofenadine vs. the second-generation antihistamines; c fexofenadine vs. placebo.

**Additional file 14: Figure S14.** Funnel plot of sedative effects: a fexofenadine vs. the first-generation antihistamines; b fexofenadine vs. the second-generation antihistamines; c fexofenadine vs. placebo.

**Additional file 15: Figure S15.** Funnel plot of CFF: a fexofenadine vs. the first-generation antihistamines; b fexofenadine vs. the second-generation antihistamines; c fexofenadine vs. placebo.

**Additional file 16: Figure S16.** Funnel plot of CRT: a fexofenadine vs. the first-generation antihistamines; b fexofenadine vs. the second-generation antihistamines; c fexofenadine vs. placebo.

**Additional file 17: Figure S17.** Funnel plot of CTT: a fexofenadine vs. the first-generation antihistamines; b fexofenadine vs. the second-generation antihistamines; c fexofenadine vs. placebo.

**Additional file 18: Figure S18.** Funnel plot of LARS: a fexofenadine vs. the first-generation antihistamines; b fexofenadine vs. the second-generation antihistamines; c fexofenadine vs. placebo.

**Additional file 19: Figure S19.** Funnel plot of VAS of drowsiness: a fexofenadine vs. the first-generation antihistamines; b fexofenadine vs. placebo.

## Data Availability

All data generated or analyzed during this study are included in this published article. Additional information may be requested directly from the study authors.

## Abbreviations

FEX: Fexofenadine
EBA: Ebastine,
LOR: Loratadine
CHL: Chlorpheniramine,
CET: Cetirizine,
BEP: Bepotastine,
EPI: Epinastine,
TER: Terfenadine,
MI: Mizolastine, BIL bilastine,
PR: Promethazine,
DI: Diphenhydramine,
OLO: Olopatadine,
HYD: Hydroxyzine,
ACR: Acrivastine,
PL: Placebo
