# Supplementary figures and images for: Antihistamine effects and safety of fexofenadine: a systematic review and Meta-analysis of randomized controlled trials
Source: BMC Pharmacol Toxicol. 2019 Nov 29;20:72. doi: 10.1186/s40360-019-0363-1 (PMC6884918; doi:10.1186/s40360-019-0363-1)

**a**

**
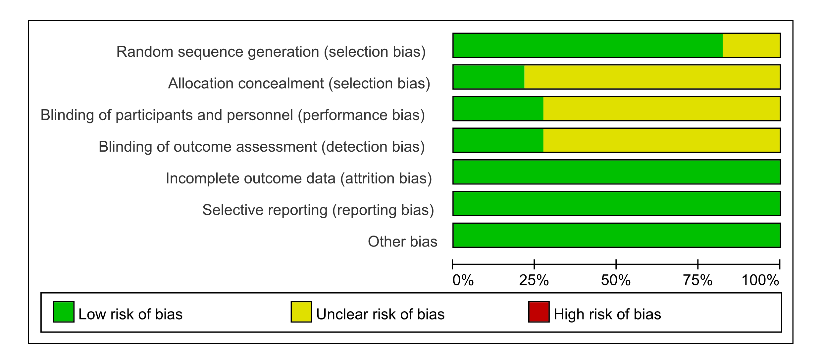
**

**b**

**
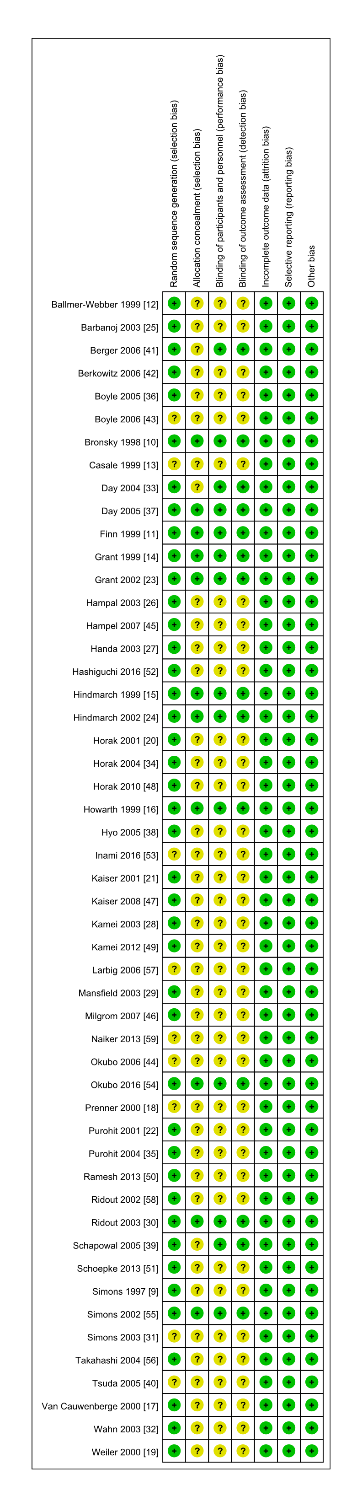
**

**Fig. S1**

Risk of bias: **a** risk of bias summary; **b** risk of bias graph.

Supplement: Supplementary file 1 — Additional file 1: Figure S1. Risk of bias: a risk of bias summary; b risk of bias graph. [file 40360_2019_363_MOESM1_ESM.docx]

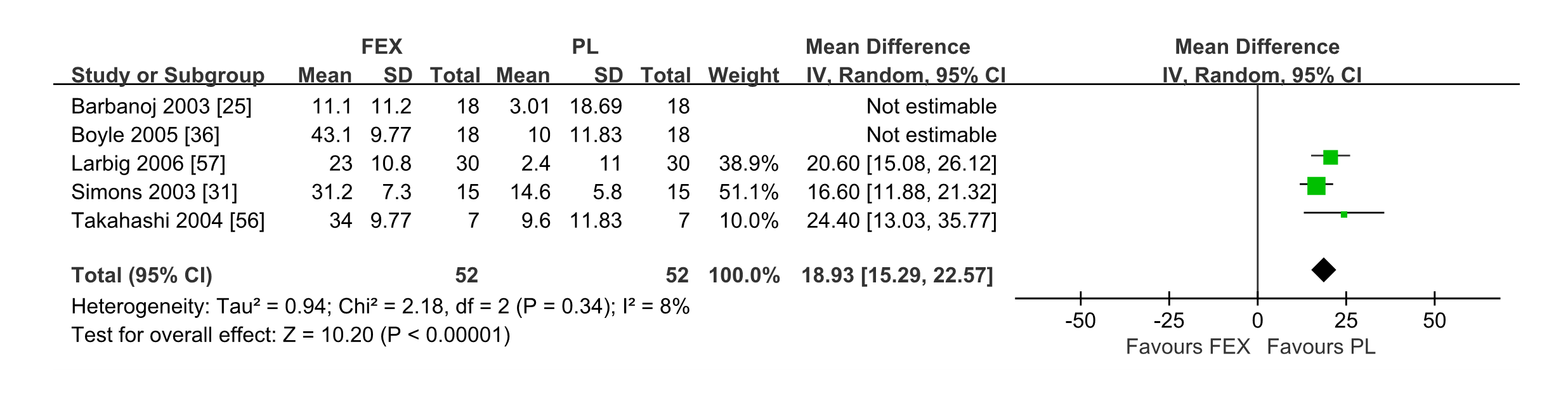

Supplement: Supplementary file 2 — Additional file 2. Figure S2. Forest plot of wheal for FEX vs. PL. [file 40360_2019_363_MOESM2_ESM.png]

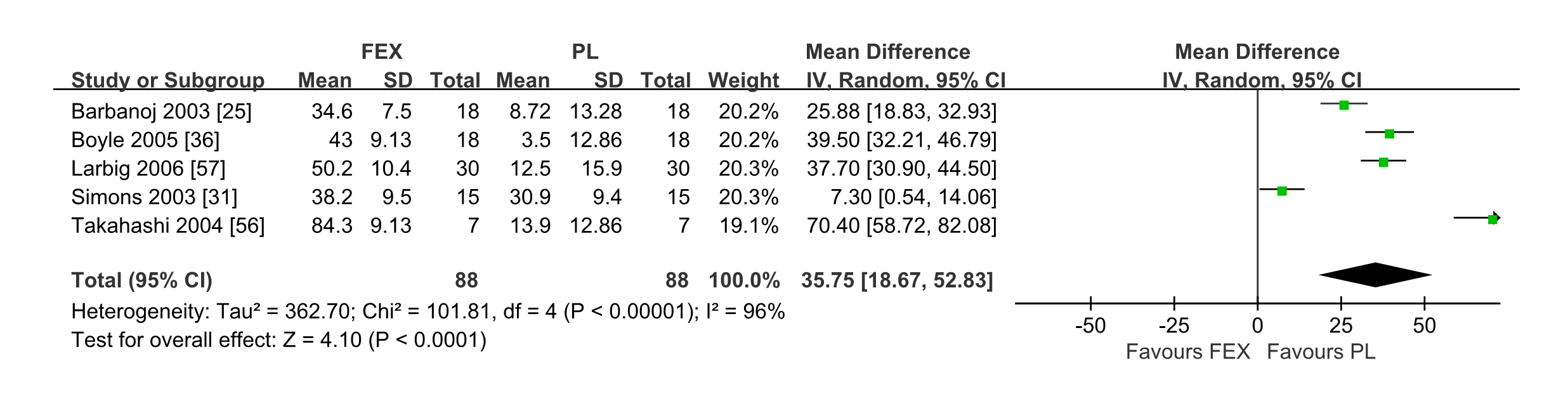

Supplement: Supplementary file 3 — Additional file 3. Figure S3. Forest plot of flare for FEX vs. PL. [file 40360_2019_363_MOESM3_ESM.png]

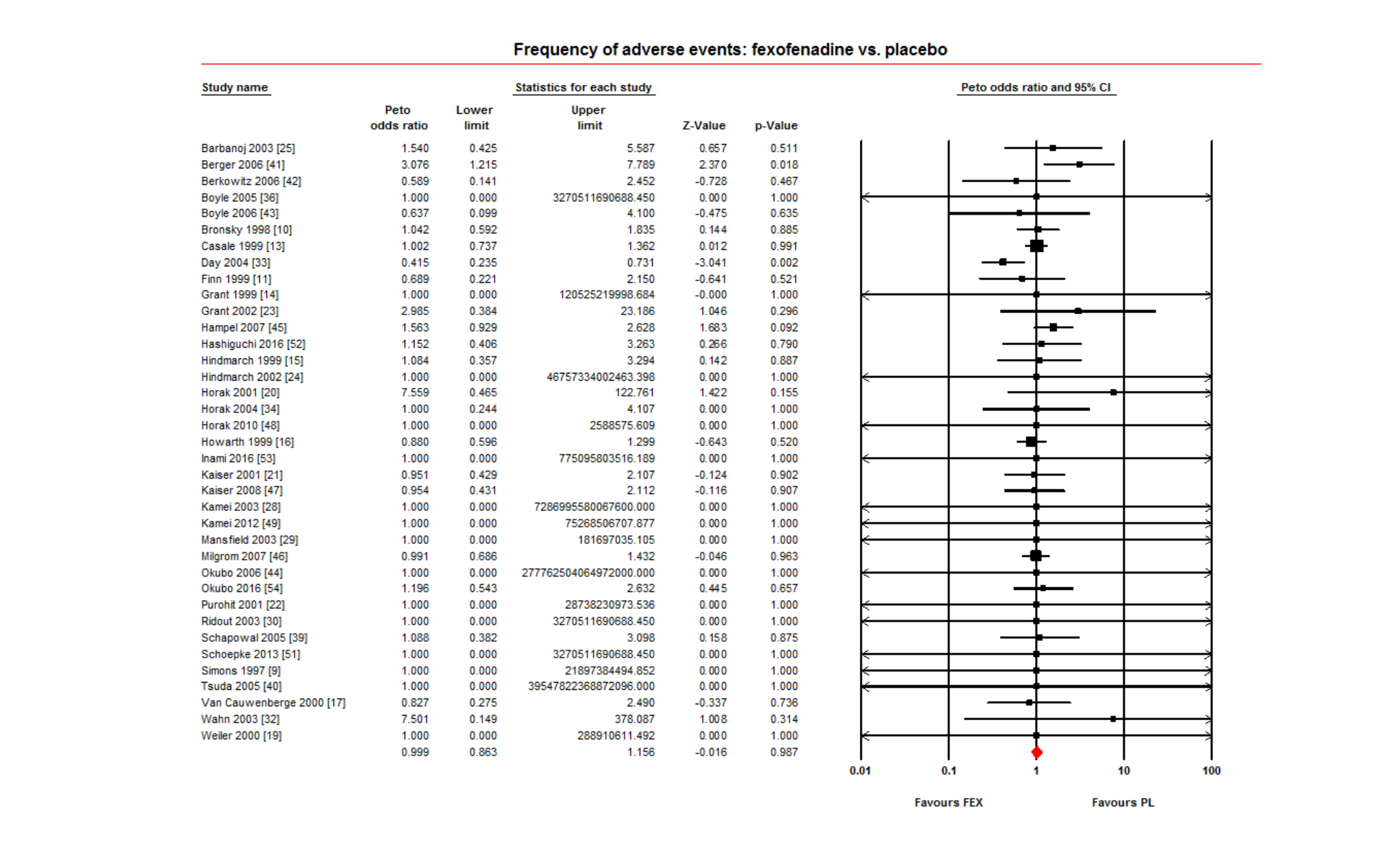

Supplement: Supplementary file 4 — Additional file 4. Figure S4. Forest plot of AE for FEX vs. PL. [file 40360_2019_363_MOESM4_ESM.png]

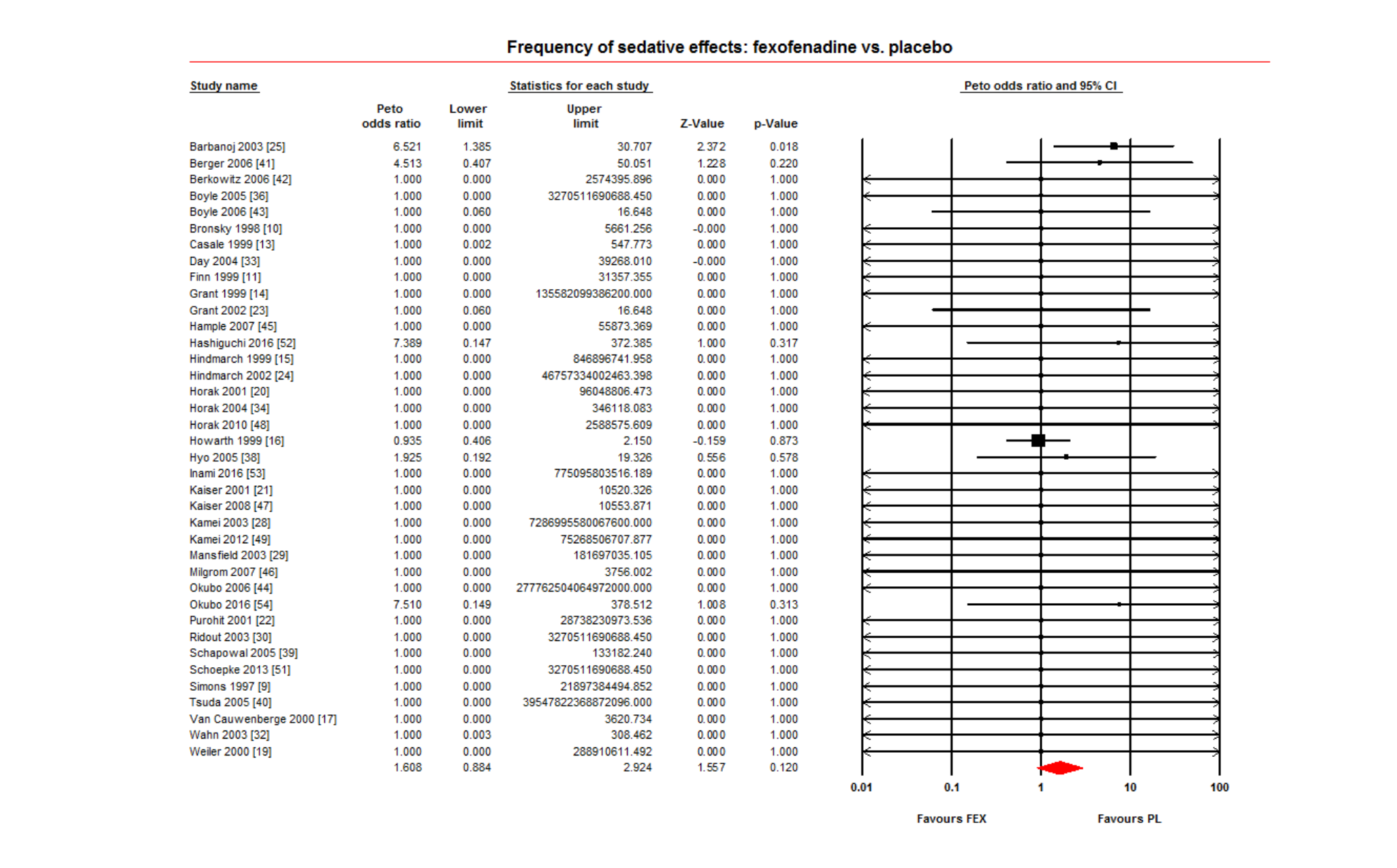

Supplement: Supplementary file 5 — Additional file 5. Figure S5. Forest plot of SE for FEX vs. PL. [file 40360_2019_363_MOESM5_ESM.png]

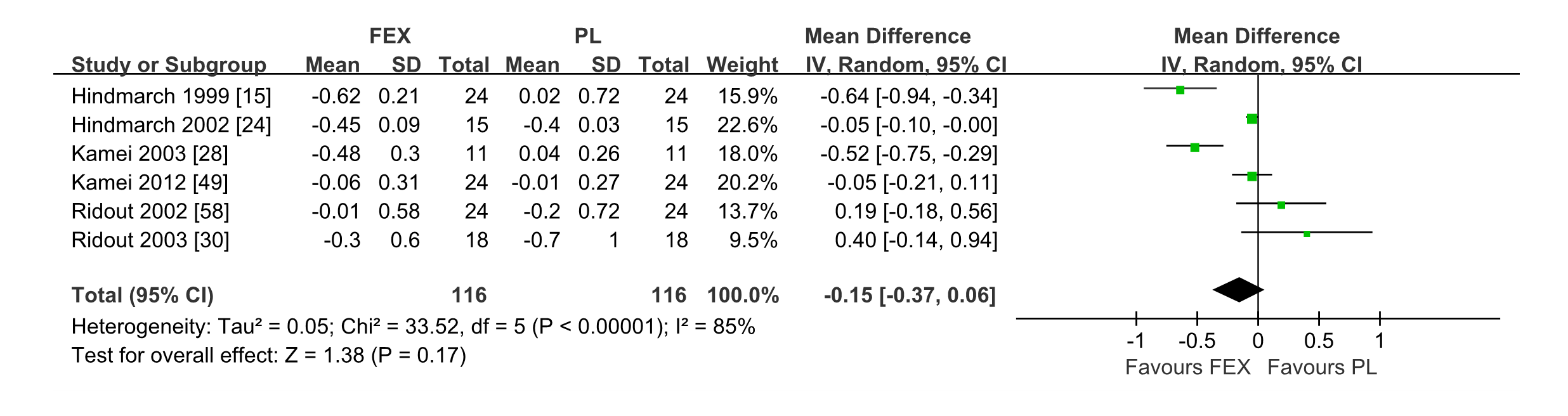

Supplement: Supplementary file 6 — Additional file 6. Figure S6. Forest plot of CFF for FEX vs. PL. [file 40360_2019_363_MOESM6_ESM.png]

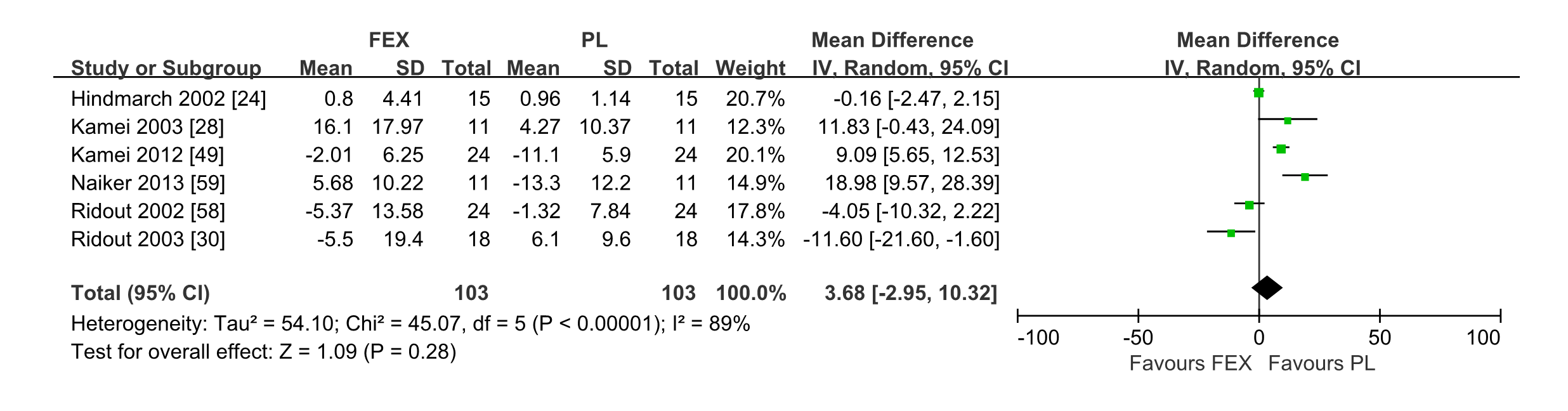

Supplement: Supplementary file 7 — Additional file 7. Figure S7. Forest plot of CRT for FEX vs. PL. [file 40360_2019_363_MOESM7_ESM.png]

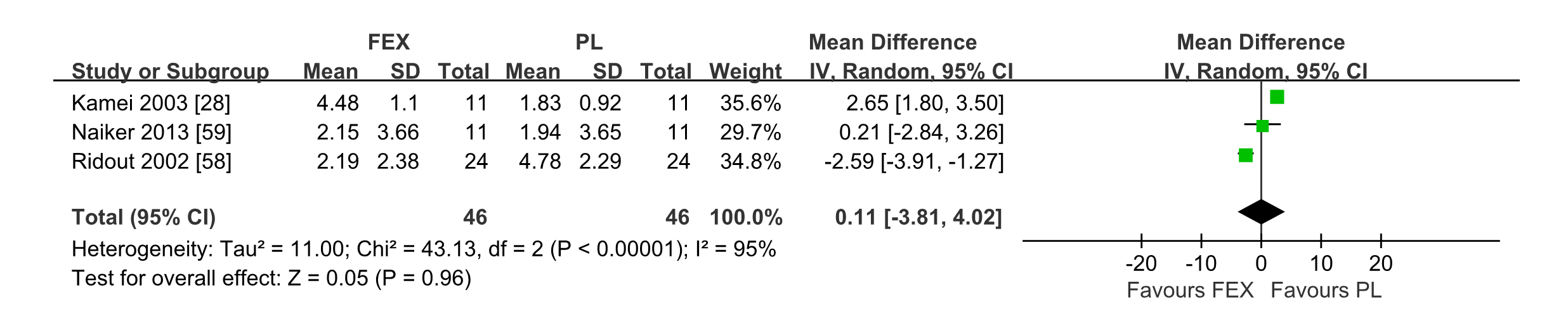

Supplement: Supplementary file 8 — Additional file 8. Figure S8. Forest plot of CTT for FEX vs. PL. [file 40360_2019_363_MOESM8_ESM.png]

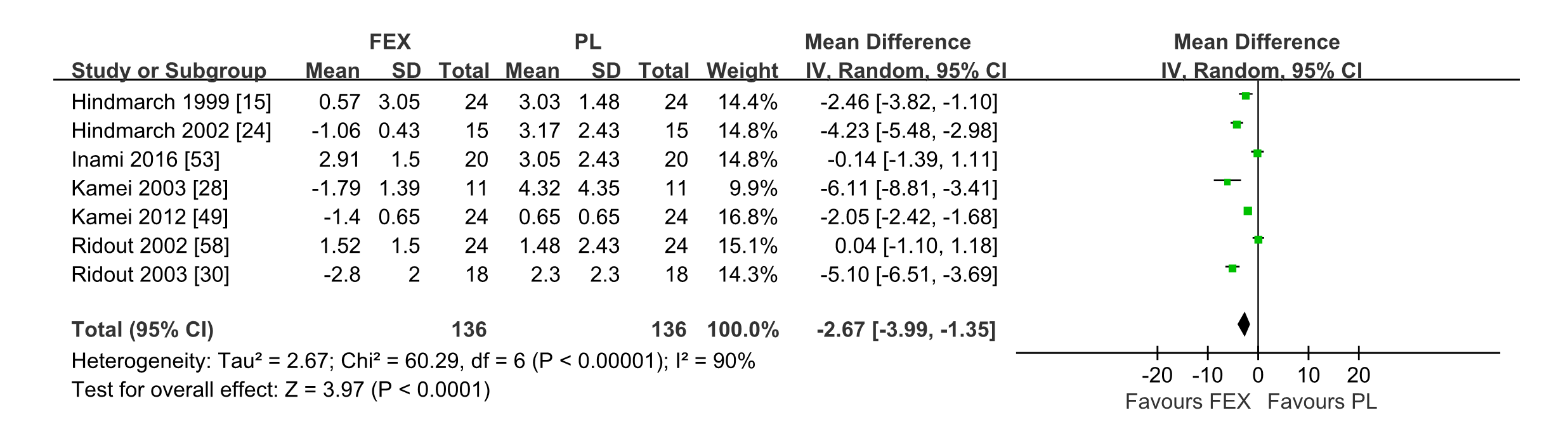

Supplement: Supplementary file 9 — Additional file 9. Figure S9. Forest plot of LARS for FEX vs. PL. [file 40360_2019_363_MOESM9_ESM.png]

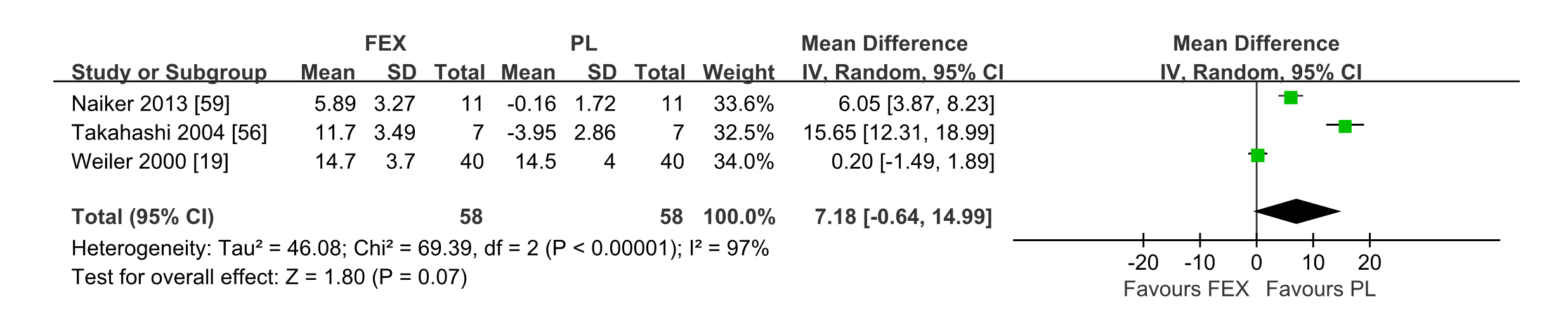

Supplement: Supplementary file 10 — Additional file 10. Figure S10. Forest plot of VAS of drowsiness for FEX vs. PL. [file 40360_2019_363_MOESM10_ESM.png]
