# Supplementary material for: Antihistamine effects and safety of fexofenadine: a systematic review and Meta-analysis of randomized controlled trials
Source: BMC Pharmacol Toxicol. 2019 Nov 29;20:72. doi: 10.1186/s40360-019-0363-1 (PMC6884918; doi:10.1186/s40360-019-0363-1)
Supplement: Supplementary file 12 — Additional file 12: Figure S12. Funnel plot of flare: a fexofenadine vs. the second-generation antihistamines; b fexofenadine vs. placebo. [file 40360_2019_363_MOESM12_ESM.docx]

**a**

**
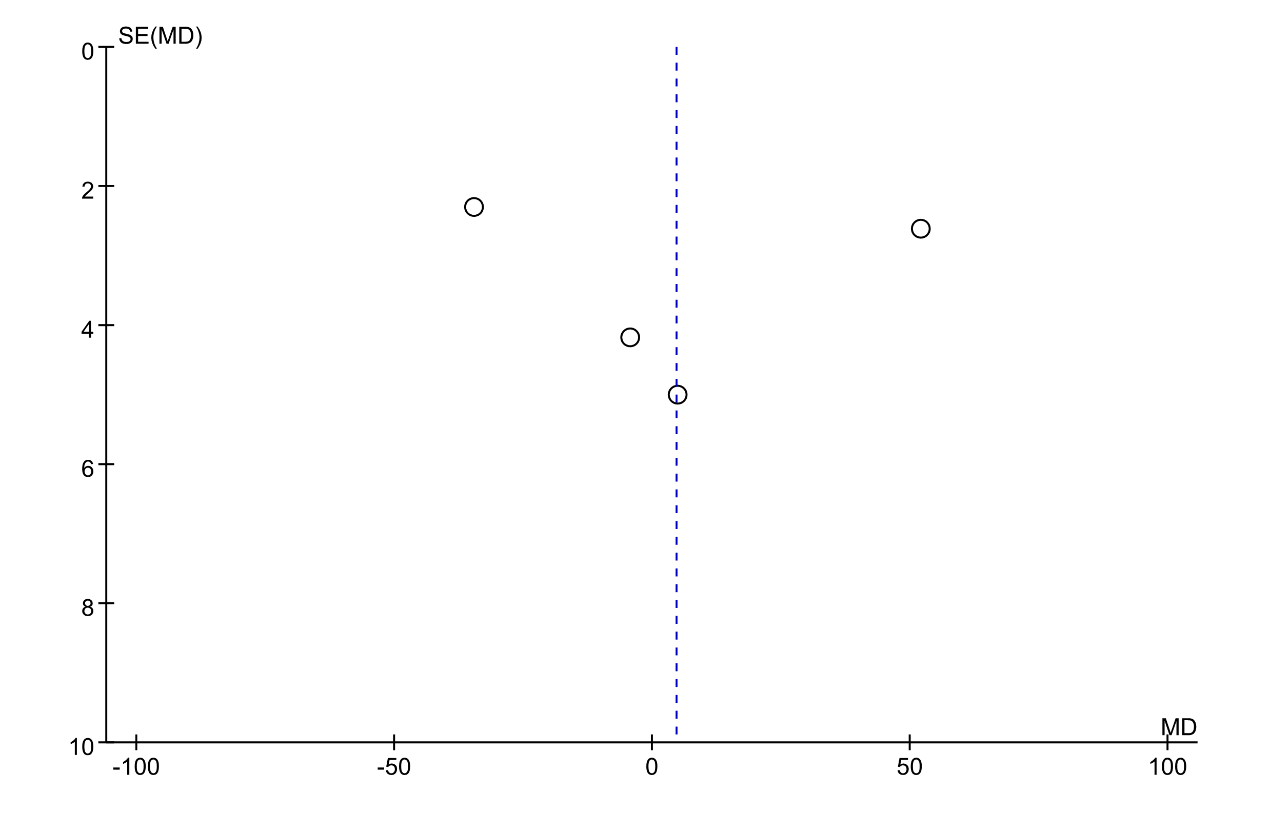
**

**b**

**
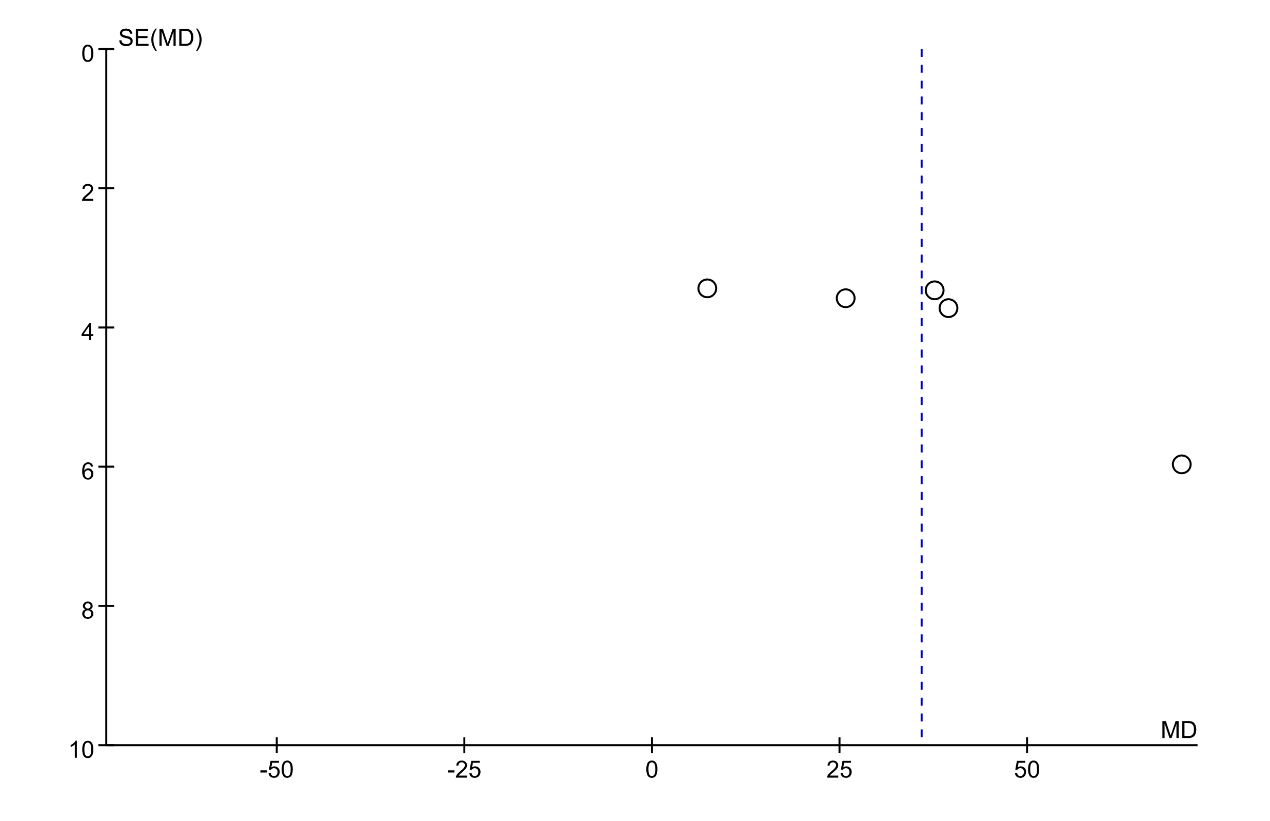
**

**Fig. S12**

Funnel plot of flare: **a** fexofenadine vs. the second-generation antihistamines; **b** fexofenadine vs. placebo.
