# Supplementary material for: Antihistamine effects and safety of fexofenadine: a systematic review and Meta-analysis of randomized controlled trials
Source: BMC Pharmacol Toxicol. 2019 Nov 29;20:72. doi: 10.1186/s40360-019-0363-1 (PMC6884918; doi:10.1186/s40360-019-0363-1)
Supplement: Supplementary file 19 — Additional file 19: Figure S19. Funnel plot of VAS of drowsiness: a fexofenadine vs. the first-generation antihistamines; b fexofenadine vs. placebo. [file 40360_2019_363_MOESM19_ESM.docx]

**a**

**
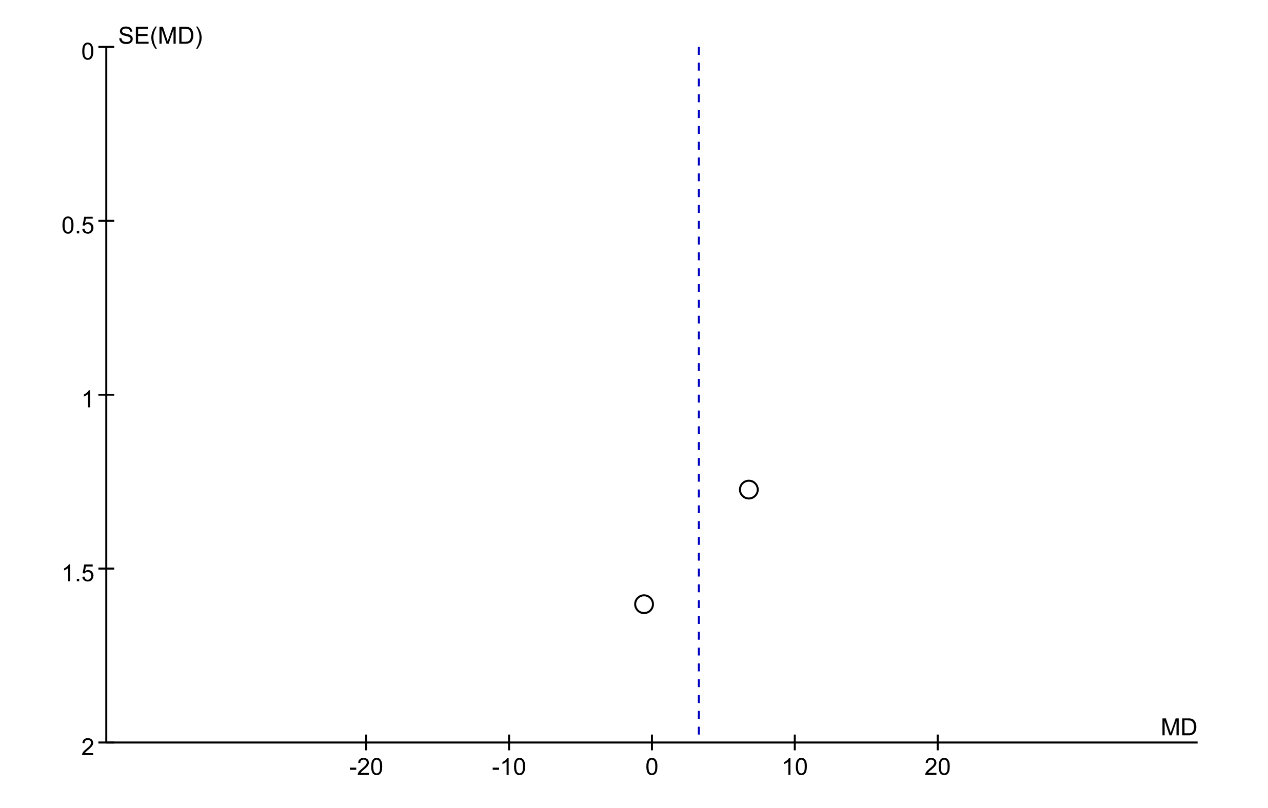
**

**b**

**
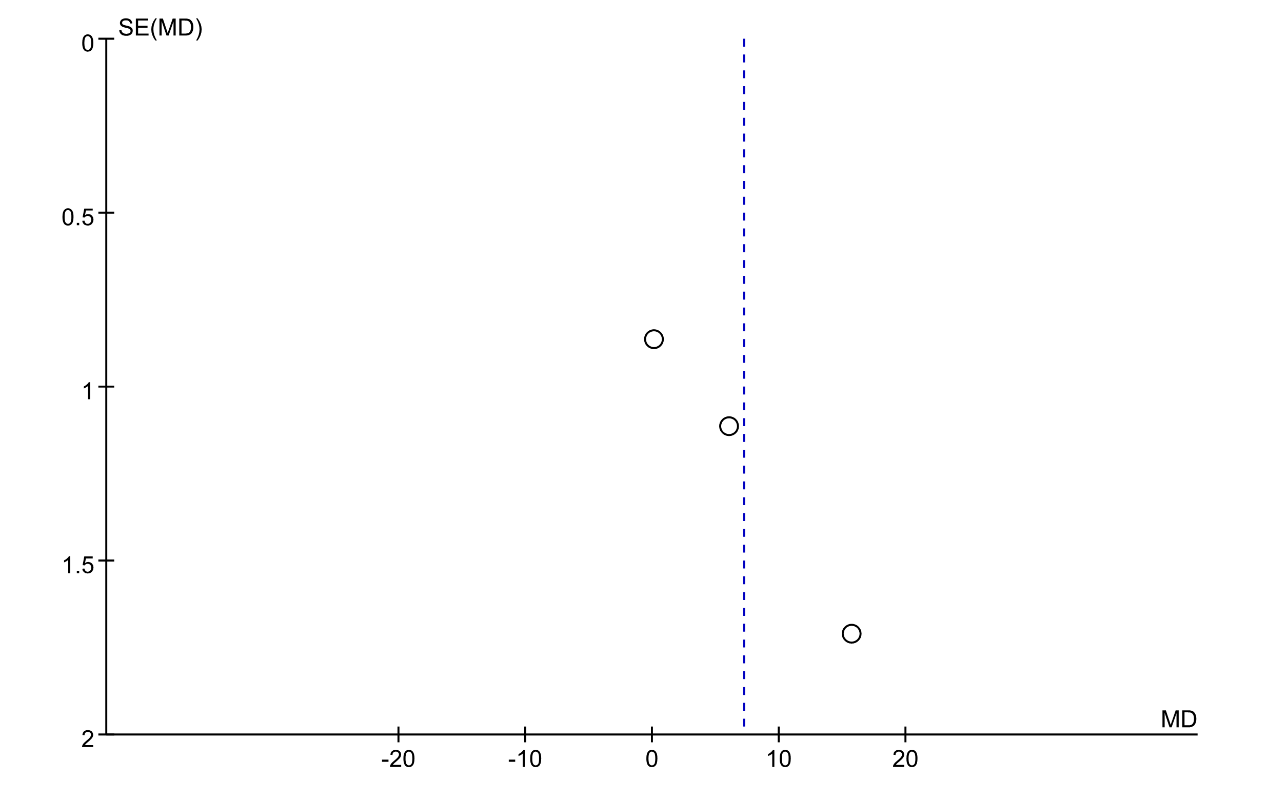
**

**Fig. S19**

Funnel plot of VAS of drowsiness: **a** fexofenadine vs. the first-generation antihistamines; **b** fexofenadine vs. placebo.
